# Non-adaptive evolutionary forces predominantly shape nucleotide composition near human transcription start sites

**DOI:** 10.1371/journal.pbio.3004063

**Published:** 2026-10-21

**Authors:** Yi Qiu, Emilie Hebraud, Fanny Pouyet, Alexander F. Palazzo

**Affiliations:** 1 Department of Biochemistry, University of Toronto, Toronto, Ontario, Canada; 2 Laboratoire Interdisciplinaire des Sciences du Numérique, Université Paris-Saclay, Gif-sur-Yvette, France; 3 Institut Agro, Rennes Angers, Rennes, France; National Centre for Biological Sciences, INDIA

## Abstract

In the human genome, mutation rates vary along genes, yet how their fine-scale structure contributes to gene evolution has remained largely unexplored. Here, we map inherited mutations at single-nucleotide resolution and uncover a striking hypermutation peak at transcription start sites (TSSs) of human protein-coding genes. This pattern is observed both in population polymorphisms and in de novo mutations from parent-offspring trios, and is dependent on transcriptional activity in testes. We also find similar hypermutation peaks at TSSs used to produce long noncoding RNAs and at intergenic RNA Polymerase II pause sites. In addition, we identify distinct mutational signatures at exon-intron boundaries and in introns. By comparing the current nucleotide content to predicted equilibrium levels, inferred from mutation and fixation rates, and by analyzing derived ancestral frequency spectrums, we detect signals that are compatible with a low level of ongoing background GC-biased gene conversion (gBGC) throughout the region and ancestral elevated gBGC activity that is centered right downstream from the TSS. Using a forward-in-time simulation algorithm, we show that the current nucleotide composition surrounding the TSS of protein-coding genes is best explained by local mutation biases coupled to ongoing and ancestral patterns of gene conversions. Our simulations allow us to infer several features of these gBGC events. Overall, these findings indicate that the nucleotide composition around TSSs is largely shaped by non-adaptive forces, particularly mutation bias and gBGC.

## Abstract 2

Mutation rates vary across human genes, but how fine scale mutational patterns influence gene evolution is poorly understood. This study shows that hypermutation at transcription start sites, together with mutation bias and GC-biased gene conversion, largely shapes nucleotide composition through nonadaptive evolutionary processes.

## Background

One striking feature of transcriptional start sites (TSSs) of human protein-coding genes is that they have high GC-content [1–4]. High GC-content in the promoter region enhances transcription [5,6], while high GC-content at the 5'end of mRNAs that have few introns promotes their efficient export from the nucleus [7–12]. It is generally assumed that such major nucleotide features of the genome are solely the product of natural selection. Despite this, new findings have indicated that non-adaptive evolutionary forces may play a significant role in shaping nucleotide content, even at protein-coding genes. For example, ancient patterns of recombination around the TSS of human protein-coding genes likely caused these regions to be enriched in GC-content [13] through the action of a strong non-adaptive force called GC-biased gene conversion (gBGC) [14]. gBGC happens at meiotic recombination initiation sites where maternal and paternal strands form mixed heteroduplexes. In these regions any mismatches due to heterozygosity are resolved in favor of G/C over A/T. This causes a bias for G/C single nucleotide polymorphisms (SNPs) to be passed down to the next generation over A/T SNPs [14]. Note that these recombination initiation events can be resolved to form either crossovers, or more frequently non-crossovers. Both processes likely promote gBGC. Although gBGC is largely directed away from the TSS in humans and rodents, most mammals have some degree of gBGC at the TSS [15]. Recombination tends to be concentrated at recombination hotspots. In most vertebrates, these hotspots are determined by PRDM9 and in its absence, recombination occurs preferentially at TSSs [16,17]. Despite this, certain alleles of PRDM9 allow for some TSS-associated recombination [18]. Because the PRDM9 gene is rapidly evolving [19], and its recognition motifs are constantly destroyed by recombination, which is mutagenic [20], TSSs likely experience dynamic cycles of GC-content change. When some TSS-associated recombination is allowed, due either to the presence of certain PRDM9 alleles, or an overall lack of PRDM9 motifs due to mutational decay, these regions rapidly gain GCs. When recombination is effectively directed away from TSSs, these regions slowly lose GCs.

Another factor that may affect the nucleotide content of TSSs is biased mutations, which can shape both adaptive and non-adaptive evolution [21,22]. TSSs are particularly mutation-prone because RNA Polymerase II accumulates at promoter-proximal pause sites [23,24], creating multiple sources of DNA damage [25]. Paused RNA Polymerase II causes local DNA unwinding and downstream supercoiling that topoisomerases must relieve by cleaving and re-ligating DNA. Failures in re-ligation generate double-stranded breaks and subsequent mutations during repair [26]. Paused RNA Polymerase II also creates DNA replication fork roadblocks; collisions between paused polymerase and DNA replication machinery produce DNA damage events, including double-stranded breaks [25]. RNA Polymerase II pausing also promotes R-loop formation that elevates local mutation rates through multiple pathways [25]. R-loops sequester the template strand in DNA-RNA hybrids, which acts as roadblocks for DNA replication forks. At the same time, R-loops leave the coding strand single-stranded and exposed for extended periods, making it vulnerable to chemical damage [27]. All of these mechanisms increase the mutation rate exactly where GC-content is highest.

These mechanisms operating at the TSS may be compounded by broader mutational processes affecting gene bodies. The DNA damage generated by either topoisomerase activity, replication-transcription conflicts, or R-loops, promotes the activity of translesion DNA polymerases, which are error-prone [28]. Whether they are attempting repairs, or simply bypassing DNA lesions, these enzymes will introduce secondary mutations that extend far away from the original damage site, potentially affecting gene bodies. Certain repair enzymes are recruited by histone modifications associated with exons, and this may lead to the accumulation of differential mutations between exons and introns [29]. Moreover, transcription elongation rates are slower over exons than introns [23] and this may further allow mutation patterns to differ in these two regions. There are also transcription-coupled DNA repair pathways that identify and repair lesions found solely on the template strand [30]. As a result, certain mutations occur more often on the coding strand and thus create strand-asymmetric mutational patterns across gene bodies.

Although most of these investigations have been conducted in somatic cells (both normal and cancerous), large scale studies indicate that transcription and DNA replication have major impacts on the rate of mutations in germ cells, thus impacting evolution [31–33]. Despite all of this, the contribution of mutational bias in shaping genomic content remains underappreciated by most molecular biologists [34].

Here we examine mutation rates with single nucleotide resolution, revealing a hypermutational signal at the TSS of protein-coding genes. We show that these peaks correlate with RNA Polymerase II-associated transcription in germ cells. Comparing equilibrium nucleotide content calculated based on human mutation rates and substitution rates, and determining how mutations have recently spread by analyzing derived ancestral frequencies, we estimate the contribution of mutational bias and gBGC in shaping nucleotide content at the TSS in humans. We then validate the effects of local mutation and fluctuations in gBGC on the nucleotide content of protein-coding genes through a simulation of gene evolution.

## Results

### The TSSs of human protein-coding genes experience elevated rates of mutations

To determine whether trends in nucleotide content around the transcriptional start site (TSS) of protein-coding genes (current average nucleotide content is shown in Fig 1A) are shaped in part by localized mutational hotspots, we identified low frequency single nucleotide polymorphisms (SNPs) in the human population using the gnomAD database [35]. Low frequency SNPs are generally evolutionarily young, having been created by recent mutational events, and hence have not experienced significant selection pressure, and thus can be used to infer the base mutation rate. The gnomAD database contains half a billion low frequency SNPs (frequency <1%), isolated from 71,702 individuals, representing one SNP for every six nucleotides. The high density of data allowed us to infer the mutation rate at single nucleotide resolution around the TSS, which we mapped using Cap Analysis Gene Expression (CAGE) sequencing. Since only germline mutations are transmitted across generations and are thus the most relevant for evolution, we used CAGE sequencing from human testes [36]. To infer local mutation rates, we defined the parameter *M*, based on the density of rare variants. The number of these low frequency variants depends not only on the mutation rate (*µ*) but also on population genetics factors such as the effective population size (*N*_*e*_), the selection coefficient (*s*) and the conversion bias due to gBGC (*b*) which affect not only the probability to observe these mutations but also their residence time in the 1% frequency bin. We therefore rescaled *M* so that its value in intergenic regions corresponds to the mutation rate, *µ*, inferred by de novo mutations as determined by parent-offspring trio sequencing compiled from recent studies (S2 Table) [37–43]. We expect that the joint effects of *N*_*e*_, *b*, and *s* are minimal for very low frequency SNPs, thus making localized values of *M* ≈ *µ*. Under the assumptions of neutrality and constant population size, the rescaling factor (calculated to be 105 using intergenic regions, see Methods under “Rare SNP mapping and derivation of *M*”) can be interpreted as the approximate average number of generations ago these SNPs appeared in the human population.

**Fig 1:**
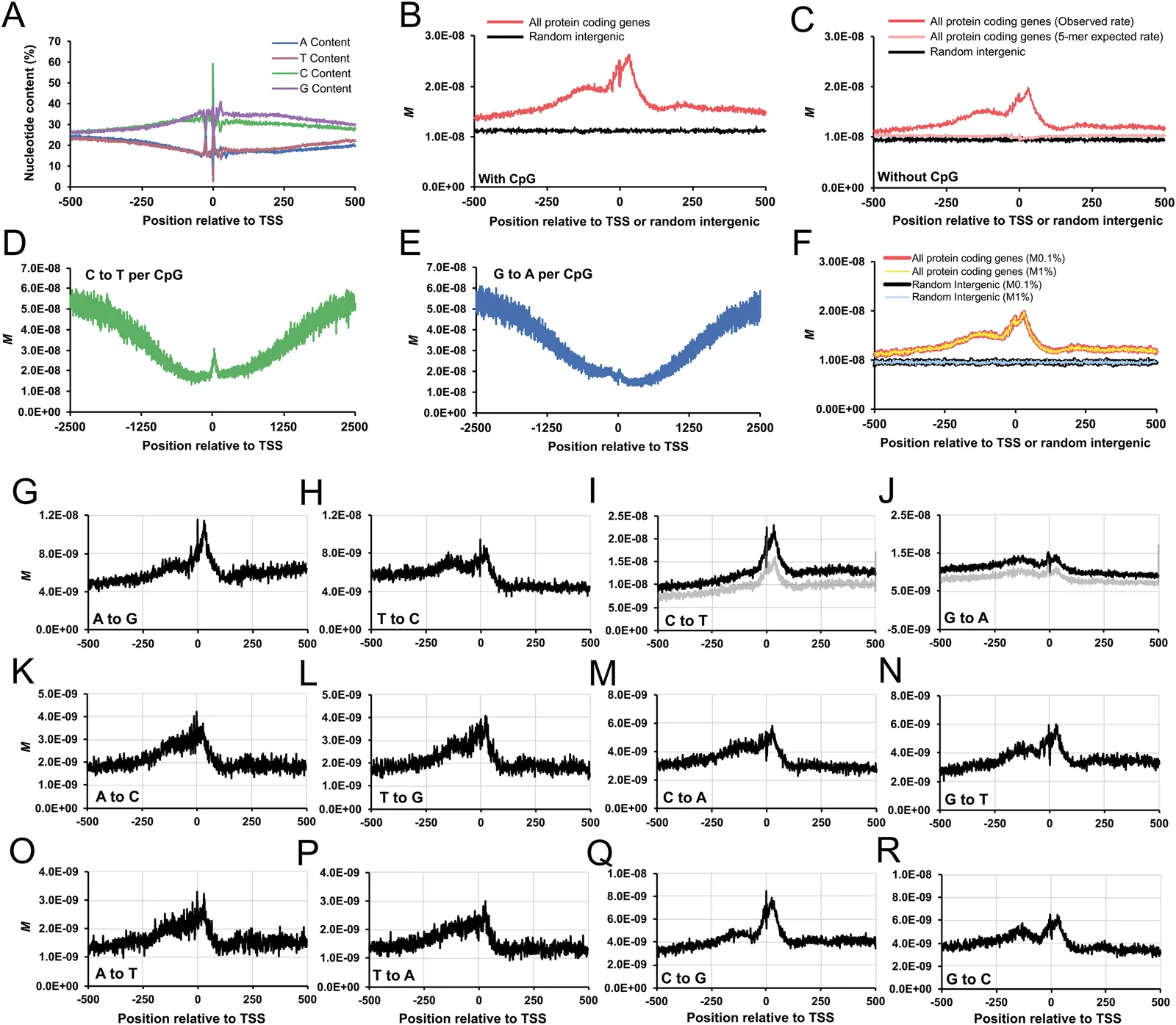
Single nucleotide resolution *M* for regions surrounding human protein-coding gene TSSs. **A**) Nucleotide content 1kb around the TSS, averaged over all human protein-coding genes and displayed at single nucleotide resolution. The TSS is positioned at 0. Underlying data can be found in S1 Data. **B**) Single nucleotide resolution of *M*_*1%*_, 1kb around all TSSs from human protein-coding genes (red) or an equivalent number of intergenic regions (black) using low frequency SNP analysis from gnomAD. Underlying data can be found in S1 Data. **C**) Single nucleotide resolution of *M*_*1%*_ for nonCpG mutations, 1kb around all TSSs from human protein-coding genes (red) or an equivalent number of intergenic regions (black) using low frequency SNP analysis from gnomAD. Single nucleotide predicted rate based on 5-mer sequence context over all TSSs from human protein-coding genes (pink). Underlying data can be found in S1 Data and S1 Data (for 5-mer context). **D-E**) Single nucleotide resolution of *M*_*1%*_, for CpG to TpG (**D**) and CpG to CpA (**E**) 5kb around the TSS of all human protein-coding genes. Underlying data can be found in S1 Data. **F**) Comparison of single nucleotide resolution of *M*_*1%*_ (red for TSS regions, black for intergenic regions) and *M*_*0.1%*_ (yellow for TSS regions, blue for intergenic regions). Underlying data can be found in S1 Data. G-R) Single nucleotide resolution of *M*_*1%*_, for each indicated mutation type 1kb around the TSS of all human protein-coding genes. For I-J, total *M*_*1%*_ is shown in black, nonCpG *M*_*1%*_ is shown in grey. Underlying data can be found in S1 Data.

Using *M,* we estimated the mutation rate surrounding the TSS of human protein-coding genes with (Fig 1B), and without CpGs (Fig 1C). Strikingly, we observed a peak in *M* in the region surrounding the TSS. In agreement with the idea that transcription is mutagenic, we observed that *M* around the TSS was higher than in intergenic regions. When we excluded CpGs, *M* was above the expected context rate (Fig 1C) as calculated using both 5-mer counts and the rate at which each 5-mer acquires low frequency SNPs in intergenic regions (see Methods under “5-mer sequence context rate calculation”). Note that the context rate was much higher when CpGs were included (S1B Fig) and this is to be expected as CpGs near the TSS are hypomethylated. Non-methylated C deaminates to U and is much more readily repaired than 5meC, which deaminates to T [44,45]. The lack of methylation around the TSS allows deaminated CpGs in these regions to be recognized and efficiently reverted back to their original state, as seen in Fig 1D and 1E, unlike CpGs in intergenic regions. The suppression in CpG mutation around TSSs is in agreement with previous substitution analyses [3].

The increase in *M* at the TSS consisted of at least two distinct peaks. The major peak was centered 25 nucleotides downstream of the TSS, while a minor one was centered at about 110 nucleotides upstream from the TSS. Furthermore, we detected a relative depression in *M* centered around 140 nucleotides downstream of the TSS followed by another shallow peak centered at 200.

To determine whether *b* or *s* was significantly altering *M*, we recalculated this metric using ultralow frequency SNPs (<0.1%), which have on average been in the population for fewer generations and experienced less selection or gBGC. The two curves (*M*_*1%*_ and *M*_*0.1%*_) were superimposable for both TSS and intergenic regions, indicating that the effects of *b* and *s* are indeed minimal (Fig 1F, also see Fig 2D described in the next section).

**Fig 2:**
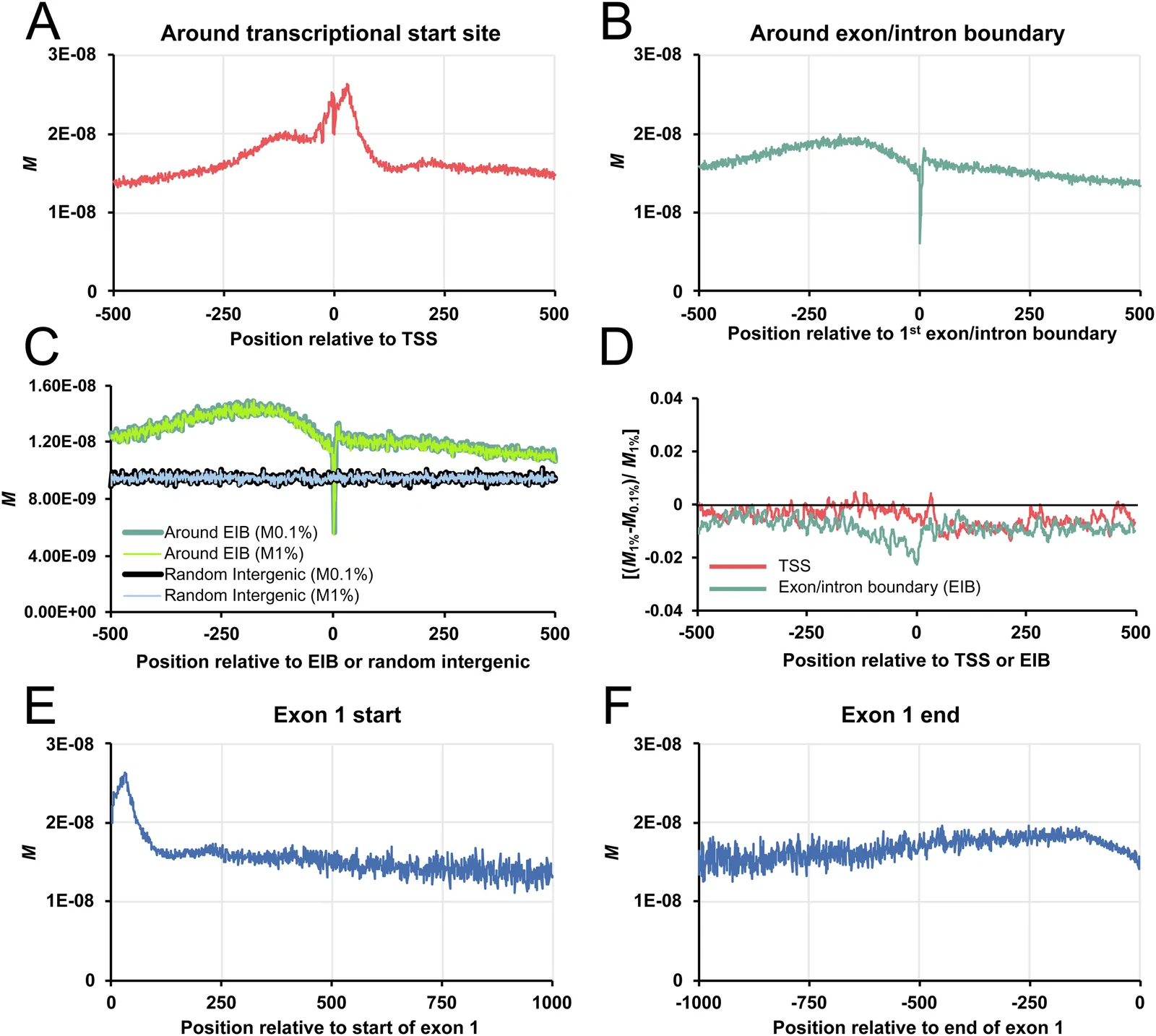
Single nucleotide resolution *M* for the first exon of human protein-coding genes. **A-B**) Single nucleotide resolution of *M*_*1%*_, over all TSSs (**A**), or the first exon-intron boundary (**B**), of all human protein-coding genes. Underlying data can be found in S1 Data. **C**) Comparison of single nucleotide resolution of *M*_*1%*_ (dark green for exon-intron boundaries or “EIB”, black for intergenic regions) and *M*_*0.1%*_ (light green for exon-intron boundaries, blue for intergenic regions). Underlying data can be found in S1 Data. **D**) Fractional change between *M*_*1%*_ and *M*_*0.1%*_ for regions surrounding the TSS and EIB. Negative numbers indicate a depletion of SNPs between 0.1% and 1% as compared to the expected value. Note that CpGs were omitted (see Methods). Also note that the greatest change occurs right at the exon-intron boundary where 5'SS motifs are located. Underlying data can be found in S1 Data. **E**) Single nucleotide resolution of *M*_*1%*_ over all first exons aligned so that the first nucleotides are positioned at 0. Underlying data can be found in S1 Data. **F**) Single nucleotide resolution of *M*_*1%*_ over all first exons aligned so that the last nucleotides (at the EIB) are positioned at 0. Underlying data can be found in S1 Data.

Next, we examined the effects of *M* on GC-content (note that by default all *M* in this study is *M*_*1%*_ unless specified). In agreement with our previous analysis of de novo mutations [13], which were inferred from parent-offspring whole genome sequencing [46], we find a net loss of Gs and Cs around the TSS (S1A Fig). Within this greater trend, there are two exceptions. First, there are fewer GC-losses in a region 23 nucleotides upstream of the TSS, a location that corresponds to the TATA box, which is relatively GC-poor. Second, there are fewer GC-losses right at the TSS and this may be due to the particular distribution of nucleotides at the + 1 and −1 positions relative to the TSS [47].

Next, we analyzed *M* for different mutation types (Fig 1G-1R) including non-CpG mutations to TpG and CpA (Fig 1I-1J grey line). The major peak (25 nucleotides downstream from the TSS) is seen in all mutation types. Interestingly, the upstream minor peak (at ~110 nucleotides upstream from the TSS) is only seen in a subset of mutation types. For example, it is not readily detectable in C to T transition mutations (Fig 1I), but is prominent with all mutations away from G (i.e., G to A, G to T and G to C, Fig 1J, 1N and 1R). We could also see a small peak near the TSS in the CpG *M* analysis (see small peaks near “0” in Fig 1D-1E) that mirrored the peak in total and non-CpG *M* graphs (S1C-S1D Fig), suggesting that these are all a product of the same process.

Looking more broadly, our measurements of *M* indicate that regions downstream of the TSS (>200 nucleotides), which are mostly intronic regions, have mutation patterns that differ from upstream regions (>200 nucleotides upstream from the TSS). For example, downstream regions have lower T to C mutations than upstream regions (Fig 1H). Notably, these patterns are not strand symmetric (i.e., regional changes in the T to C mutation rate are not mirrored in the A to G mutation rate – compare Fig 1H to 1G). In the absence of selection, this is expected to produce strand asymmetry, also known as skew. This is consistent with the idea that the nucleotide distributions in human introns are due to subtle differences in the nucleotide mutation or repair rates between the template and coding strands [3,12,31,48].

We also analyzed somatic mutations around the TSS of protein-coding genes based on the SomaMutDB (S1E-S1F Fig). We excluded datasets from samples with DNA damage repair defects since these would cause additional biases. In this dataset, we also observe a hypermutational peak from somatic mutations around the TSS. However, we only observe one prominent peak centred ~30 nucleotides downstream of the TSS, mirroring the main peak in *M* derived from germline mutations (compare S1E-S1F Fig to Fig 1B-1C).

Using 25 base pair windows spanning the 1kb region surrounding the TSS of protein-coding genes or random intergenic sequences, we assessed SBS mutational signatures based on rare SNPs (<1%) according to COSMIC-SigProfiler [49,50]. While intergenic regions had only the SBS5 signature, which is a background clock-like signature consistent with previous analyses of germline mutations [51], the regions directly surrounding the TSS of protein-coding genes were dominated by other signatures, indicating an enrichment of mutational processes associated with transcription-associated damage and DNA repair (S2A Fig). In particular, we observed an enhancement for SBS3, SBS12, SBS16, SBS26, SBS30, SBS37, SBS39 and SBS93. Further away, SBS5 is more dominant but appears in combination with these signatures and others (e.g., SBS19, SBS32 and SBS40c).

Overall, these signatures are linked to distinct DNA repair pathways, including defective homologous recombination DNA damage repair (SBS3) [52], nucleotide excision repair on the coding strand of transcribed regions (SBS12 and SBS16) [53], defective DNA mismatch repair (SBS26) [54], defective base excision repair (SBS30) [55], double-stranded DNA break repair through non-homologous end joining (NHEJ) or microhomology-mediated end joining (SBS39) [56], and polymerase theta-mediated end joining (TMEJ) double-stranded break repair (SBS40c) [57]. SBS3, SBS12, SBS16, SBS30, and SBS40c were previously reported near TSSs for germline mutations [33]. Note that SBS39, which is enriched in C to G mutations, and associated with homologous recombination deficiencies [58], was also found in Cortés Guzmán et al. [33] and resembles the “germline component 8” signature detected in a separate study [32]. Although SBS37 has an unknown etiology, it is similar to SBS12 (S2B Fig). Other signatures, such as SBS19 and SBS32, which are enriched in C to T mutations, resemble SBS30 (S2B Fig).

Together, these results support the idea that TSS regions are enriched for multiple mutational processes linked to replication asymmetry and DNA damage repair.

### The main mutational hotspot downstream of the TSS occurs within the first exon

To determine where the main mutational hotspot lies, with respect to other gene features, we reanalyzed *M* for all mutations (Fig 2A) and the 12 individual mutation types (S3 Fig), with respect to exon and intron boundaries.

When protein-coding genes were lined up so that the boundary between their first exons and first introns was aligned (exon-intron boundary, EIB), we could see a dip in *M* right at the boundary (Fig 2B). This is likely due to the fact that mutations in many 5′ Splice Sites (5'SS), which recruit the spliceosome to the 5'end of the intron, are under high levels of selection [59], and thus under-represented in the rare SNP pool. Upstream of the EIB, *M* gradually increases to form a broad peak that is likely due to the spreading out of the main peak in *M* that we previously mapped 25 nucleotides downstream of the TSS (see Fig 2A). Within the intron we observed a steady decrease in *M* (Fig 2B), although this seems to be true for some classes of mutations (for example C to A, S3G Fig column 4) but not others (for example A to G, S3A Fig column 4).

To gauge how selection is affecting these regions, we recalculated *M* for ultrarare SNPs (<0.1% frequency) to look for changes. Again, the two graphs were almost completely superimposable (Fig 2C). When we measured the fractional difference between *M*_*1%*_ and *M*_*0.1%*_, the biggest dip (amounting to a change in 2%) was right at the exon-intron boundary where the 5'SS is found (Fig 2D). This change was larger than at any other region, including around the TSS (Fig 2D) and is in agreement with the idea that 5'SS motifs are under negative selective pressure. Other differences could be seen upstream of the exon-intron boundary indicating some selection in these areas, which encompass coding regions in the first exons. There also was a slight depression downstream of the exon-intron boundary (EIB) and this may indicate that some intronic sequence is under selection, or is subject to background selection as these regions are linked to nearby coding sequences.

Next, we lined up all first exons while excluding all intronic sequences and recalculated *M* (“Exon 1 start”). We observed a clear signal at 25 nucleotides downstream from the TSS (Fig 2E). This could also be seen for all mutation types (S3A-S3L Fig column 2), suggesting that the main peak in *M* was not due to the elevation of a particular type of mutation. At longer distances away from the TSS, we see a drop in *M* that is similar to what is seen in introns (compare the slope in Fig 2E, of nucleotides >250 downstream of the exon 1 start, to the slope of Fig 2B, of nucleotides downstream of the exon-intron boundary). When exons were lined up so that the last nucleotides of exon 1 are aligned (“Exon 1 end”, all alignments start at 0 on the right side of the *x-axis* in Fig 2F), we saw a decline in *M* that starts at 150 nucleotides upstream of the exon-intron boundary and continues to fall as one approaches the boundary (Fig 2F). Again, this is true for all mutation types (S3 Fig, column 3).

From this analysis, we concluded that the overall patterns of *M* in the exons are more complicated than those seen in the introns. Some patterns are mutation type specific, others are found across all mutation types.

### The TSS-associated mutational hotspot is seen in de novo mutations

As described above, *M* may be affected by selection and gBGC, although this seems minimal (Fig 2D). To verify our results, we analyzed the de novo mutations from parent-offspring trio sequencing studies (a genome-wide total of ~1.08x10^6^ single nucleotide mutations compiled over several published studies, see S2 Table) averaged over all genes. As was seen for *M* (Fig 3A, red line, also see the statistical comparison of *M* in TSS versus intergenic regions displayed in Fig 3B, red bar), we observed the two major peaks around the TSS of protein-coding genes, regardless of whether we included (Fig 3C) or excluded CpGs (S4A Fig). As with *M*, this rate was above the expected context rate (S4A Fig) and significantly above the intergenic rate (see black lines in Figs 3C and S4A, also see statistical analysis in Fig 3D, red bar).

**Fig 3:**
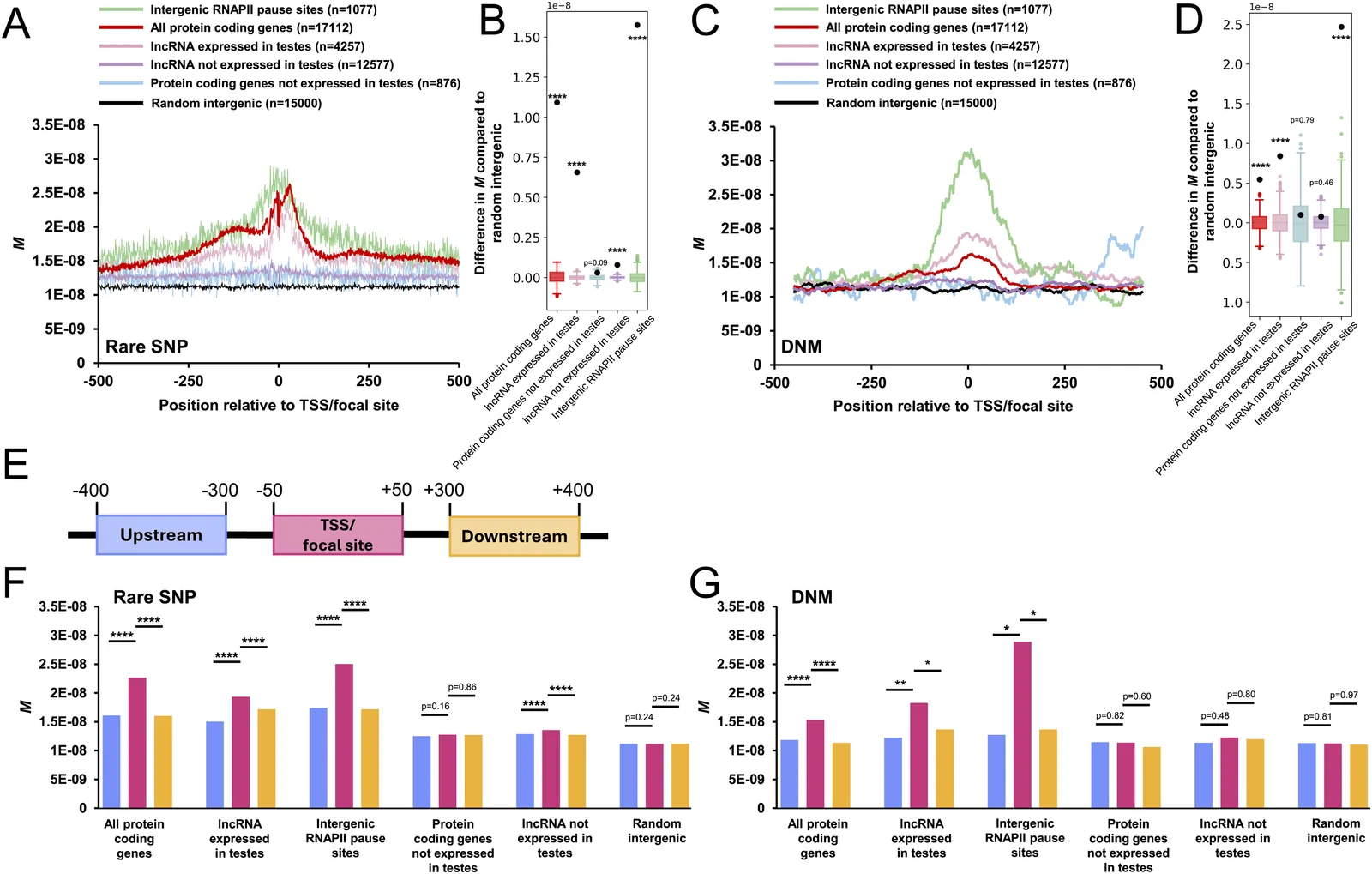
Hypermutation signals around human protein-coding gene TSSs correlate with transcription in the testes. **A**) Single nucleotide resolution of *M*_*1%*_, over various indicated regions from the human genome. **B**) Permutation test results comparing *M*_*1%*_ for a 100 bp window around the 0 point for various indicated regions, against random intergenic regions. Distribution of differences from 1,000 randomized permutations are displayed in box and whisker plots. Actual differences are plotted with black dots with *p* values indicated. Underlying data can be found in S1 Data. **C**) Mutation rates, averaged over all indicated regions from the human genome, based on de novo mutations as inferred by parent-offspring trio sequence analysis. Rates are plotted as a sliding window of 100 nucleotides. Underlying data can be found in S1 Data. **D**) Permutation test results comparing mutations rates as inferred by parent-offspring trio sequence analysis for a 100 bp window around the 0 point averaged over various indicated regions, against random intergenic regions. Distribution of differences from 1,000 randomized permutations are displayed in box and whisker plots. Actual differences are plotted with black dots with *p* values indicated. Underlying data can be found in  S1 Data. **E**) Diagram depicting analyzed zones used in (**F-G**) to determine the presence of a mutational peak. Underlying data can be found in S1 Data. **F**) *M*_*1%*_ over 100 bp windows as indicated in (**E**) for various indicated regions. *p* values between the center and upstream or downstream regions are indicated. Underlying data can be found in S1 Data. **G**) Mutation rates, averaged over 100 bp windows as indicated in (**E**) for various indicated regions. *p* values between the center and upstream or downstream regions are indicated. *: *p* < 0.05; **: *p* < 0.01; ***: *p* < 0.001; ****: *p* < 0.0001. Underlying data can be found in S1 Data.

We noted that the peak in de novo mutations was smaller than what was measured for *M* (see Discussion). However, despite this, we concluded that this second analysis validated our previous finding that there was an excess of mutations around the TSS.

### Mutational spectra associated with protein-coding TSSs are absent in genes not expressed in the testes

The association of distinct patterns of *M* around the TSS supports the notion that the act of transcription may be mutagenic, as has been documented in normal and cancerous somatic cells [25]. If this were true, and since only germline mutations are transmitted, the patterns of *M* that we observe are not expected to be present in genes that are transcriptionally silent in the germline or in gametes. Moreover, since about 70% of all mutations occur in the paternal chromosomes [46], we hypothesized that these mutations would be more pronounced in DNA regions that are undergoing transcription in the testes.

In agreement with our predictions, *M* compiled from genes not expressed in testes lacked the hypermutation peak (Fig 3A, compare red and blue lines, see statistical analysis for *M* at the TSS in Fig 3B, red and blue bars). This was true regardless of whether CpGs were included (Fig 3A) or excluded (S4C Fig). Unlike what we observed for all protein-coding genes, *M* compiled from genes not expressed in testes is similar to the predicted context rate (compare S4D–S4E Fig). Additionally, when we assessed the SBS mutational signatures associated with the TSS of genes not expressed in testes using rare SNPs (<1%), we found that SBS5 was the predominant signature (S2A Fig). Despite this, we observe some of the main signatures generally associated with the TSSs of protein-coding genes, but at low levels (S2A-S2B Fig). This could be due to either a low level of transcription in testes (below our cutoff), transcription in post-zygotic cells, or noise due to the low number of mutations in each bin (due to the paucity of genes in the non-expressed in testes pool, see Fig 3A legend) that reduces the signal.

When we analyzed de novo mutations in protein-coding genes that were not expressed in testes, we did not observe any obvious peak (Fig 3C-3D), although the data was quite noisy due to the low number of genes in that set. To increase the amount of data we looked at 100 nucleotide windows (+/- 50 nucleotides from the TSS, −300 to −400 nucleotides for upstream sequences and 300–400 nucleotides for downstream sequences; Fig 3E). Bona fide peaks should have elevated metrics at the TSS compared to nearby regions. For both *M* (rare SNP, Fig 3F) and de novo mutations from parent-offspring sequencing (DNM, Fig 3G), protein-coding genes in general had a hypermutation peak signal associated with the TSS (Fig 3F-3G). This peak was also seen when *M* was calculated for all individual mutation types (S5 Fig). In contrast, protein-coding genes not expressed in testes had no difference for overall mutations (Fig 3F-3G) or for any specific mutation type (S5 Fig).

From this data we conclude that genes not expressed in testes lack the mutational signal seen at the TSS of most protein-coding genes.

### Mutational spectra associated with protein-coding TSSs are also seen in regions expressing lncRNAs in testes

To further validate the idea that these trends in *M* are due to transcription-based mutagenesis, we examined TSSs associated with regions producing annotated lncRNAs in testes. Note that most lncRNAs are transcribed by RNA Polymerase II. Remarkably, these regions had the same overall trend in *M* as protein-coding genes, regardless of whether CpGs were included (see pink lines Fig 3, and statistics in Fig 3B pink bar) or excluded (S4C Fig). Again, this inferred hypermutational peak was above the predicted mutation rate based on 5-mer context (S4F Fig). When the mutation rate around these TSSs was examined using de novo mutations from parent-offspring sequencing, we again observed a significant difference when these were compared to intergenic regions (Fig 3C pink line, Fig 3D pink bar). The peak was significant when compared to upstream and downstream sequences in both the *M* analysis and de novo mutations (Fig 3F-3G). When we examined *M* for individual mutation rates, we could detect the main hypermutation peak for each mutation type (S5 Fig).

We then examined *M* around TSSs of regions transcribed into annotated lncRNAs that are not expressed in testes. The *M* in these regions was mostly flat regardless of whether (Fig 3A) or not (S4C Fig purple lines) CpGs were included, although there was a minute peak that was statistically higher than in intergenic regions (Fig 3B purple bar, also Figs 3F and S4G). Note that unlike the case for protein-coding genes, the number of lncRNAs not expressed in testes is high enough to detect statistical differences. We suspect this minute but statistically significant peak might be due to either low levels of expression below the threshold, or due to expression in post-fertilized germline cells. When we examined de novo mutations from parent-offspring trios there was no significant peak present (Fig 3C purple line, Fig 3D purple bar, Fig 3G). These regions had *M* similar to what was predicted by the 5-mer context (S4G Fig).

Using rare SNPs (<1%), we calculated the SBS signatures 1kb around lncRNA TSSs. We observed many of the same signatures that we had previously seen in protein-coding genes (S2 Fig), although these signatures were reduced in proportions and replaced with SBS5. For TSSs of lncRNAs that are not expressed in testes these signatures were almost entirely gone and replaced with SBS5.

From the many resemblances that we have documented between TSSs for regions transcribed into lncRNAs and protein-coding genes, we concluded that both regions undergo the same mutational biases to different degrees.

### Intergenic RNA Polymerase II pause sites are also associated with hypermutation

One of the main reasons that transcription is thought to be mutagenic is that RNA Polymerase II tends to pause just downstream of the TSS, which can cause mutagenesis by several mechanisms [25].

With this in mind, we analyzed *M* surrounding intergenic RNA Polymerase II pause sites, as mapped in testes. Note that these pause sites were filtered to exclude any that started near a protein-coding gene or a region transcribed into lncRNA to avoid using alternative TSSs of bona fide genes. As such, these sites likely represent non-functional regions that evolved motifs that have biochemical activity through drift [34, 60–62]. Unlike TSSs, we do not have single nucleotide resolution for the exact start site and we do not have strand-specificity, thus all sites were analyzed on the + strand and represent a mixture of sequence from template and coding strands (with respect to RNA Polymerase II). Remarkably, intergenic RNA Polymerase II pause sites have *M* values and de novo mutation rates that exceed what we measured at protein-coding or lncRNA TSSs (Fig 3A-3G). The same inferred hypermutational signal is also observed at these sites when only non-CpG SNPs are analyzed (S4B-S4C Fig). As with protein-coding gene TSSs and lncRNA TSSs, *M* for RNA Polymerase II pause sites was higher than what was predicted based on 5-mer sequence context (S4H Fig). This was also true for de novo mutations (S4B Fig). Note that the pattern of *M* around intergenic RNA Polymerase II pause sites appears symmetrical (Fig 3A), which is expected given that we do not have strand information.

When we examined the SBS signatures around RNA Polymerase II pause sites, we observed mostly the same signatures as seen in protein-coding genes and lncRNAs (S2 Fig).

From this data, we conclude that the presence of paused RNA Polymerase II in the testes genome is sufficient to promote an increase in inherited mutations. These mutations appear to have similarities to those that appear around the TSS of protein-coding genes.

### Equilibrium nucleotide contents suggest ongoing background gBGC and ancestral TSS-associated hotspots of gBGC

We used *M*, to calculate the expected equilibrium level for all four nucleotides at each position in the vicinity of human protein-coding TSSs and compared these to the observed nucleotide levels (Fig 4A-4D). To simplify our calculations, we only examined non-CpG positions and non-CpG *M*. We also calculated the expected equilibrium nucleotide level based on substitution rates as calculated from comparing human and chimpanzee sequences, using gorilla as an outgroup. We plotted equilibrium levels from both human and chimpanzee. Although they were both noisy due to the paucity of data, they resembled each other (Fig 4A-4D, blues lines). As expected from our previously published results [13], which indicated that the GC-peak is under decay at a rate consistent with neutral evolution, the current levels of G and C in the vicinity of the TSS are significantly higher than the predicted equilibrium levels based on the substitution rate (Fig 4A-4D, compare black and blue lines). Using gorilla as an outgroup to derive substitution rates raises potential issues with ancestral allele polarization. To verify our results, we repeated the substitution analysis using the inferred ancestral sequence from ENSEMBL that was derived from a probabilistic reference-free ancestral state reconstruction [63]. Equilibrium levels calculated from ancestral sequence substitution rates (S6A-S6D Fig) were nearly identical to the substitution rates from our trio substitution analysis (Fig 4A-4D), suggesting no major issues with polarization.

**Fig 4:**
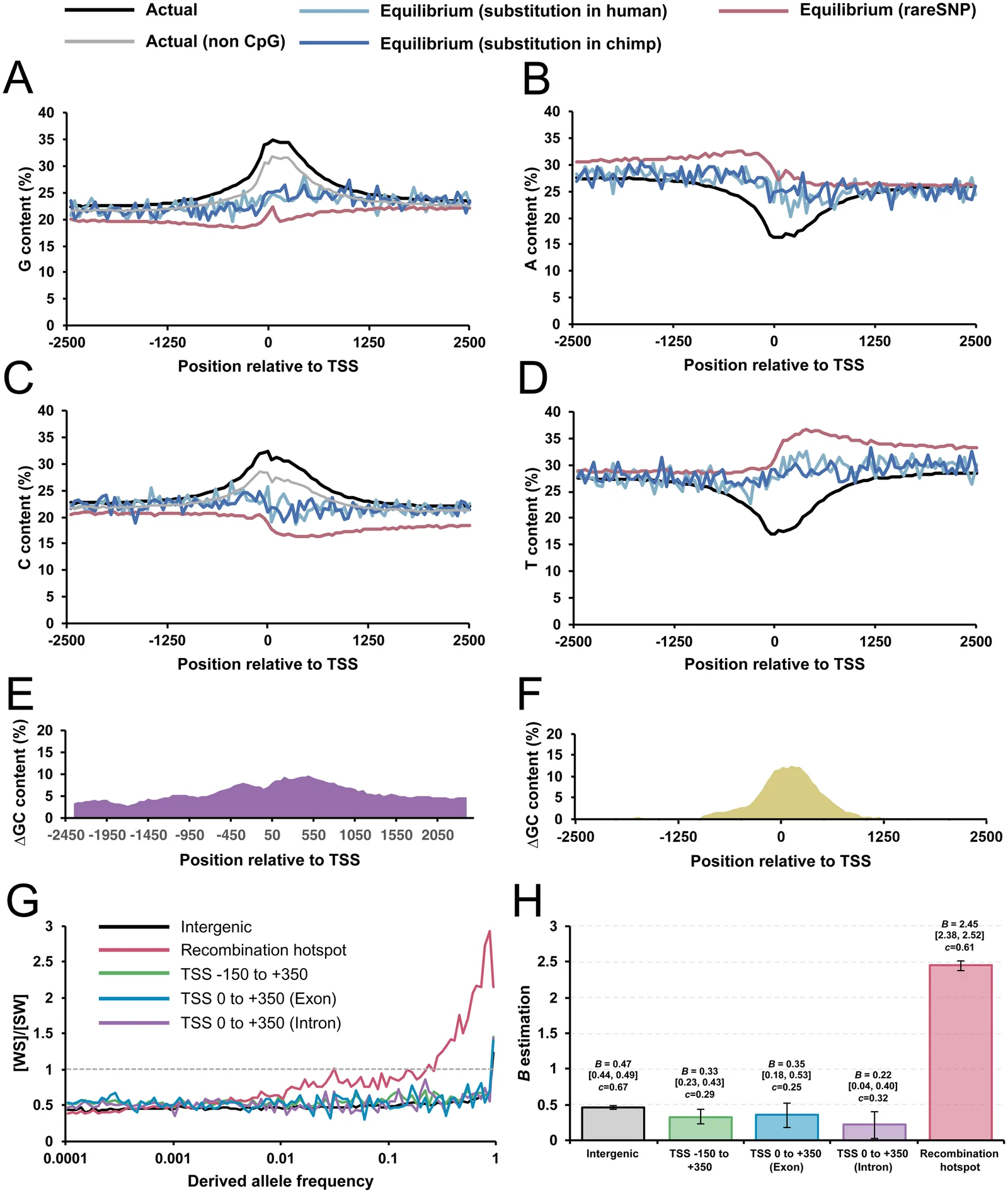
Equilibrium analysis and derived allele frequency spectra reveal ancestral, not ongoing, TSS-associated gBGC. **A-D**) The observed (black lines) nucleotide content and predicted equilibrium nucleotide levels based on either *M*_*1%*_ (pink lines) or substitution rates inferred from human-chimpanzee-gorilla trio analysis (blue lines) for regions surrounding the TSS of human protein-coding genes. All plots use bins of 50 nucleotides. Underlying data can be found in S1 Data. **E**) Difference in GC-content between predicted equilibrium levels based on substitution rates (averaged between human and chimpanzee) and *M*_*1%*_ for regions surrounding the TSS of human protein-coding genes. The values use a sliding window of 250 base pairs. Note that the difference is uniform across the region, suggesting that background gBGC is acting throughout this region. Underlying data can be found in S1 Data. **F**) Difference in GC-content between current nucleotide content in the human genome and predicted equilibrium levels based on substitution rates (averaged between human and chimpanzee rates) for regions surrounding the TSS of human protein-coding genes. The values use a sliding window of 250 base pairs. Note that the difference is most pronounced in a peak centered just downstream of the TSS of the graph, suggesting that TSS-associated gBGC occurred before the divergence of humans and chimpanzees. Underlying data can be found in S1 Data. **G**) Ratio of weak-to-strong (WS) over strong-to-weak (SW) variants across derived allele frequency (DAF) bins for intergenic regions (black), recombination hotspots (pink), and the TSS −150 to +350 window partitioned into total, exonic, and intronic positions (green, blue, purple). Variants were polarized to ancestral and derived alleles using Ensembl’s primate ancestral sequence. Underlying data can be found in S1 Data. **H**) Maximum-likelihood estimates of the population-scaled gBGC coefficient *B* for each region, with 95% confidence intervals from profile likelihood. *B* was fit jointly with a baseline mutation-bias parameter c that captures region-specific differences in mutation rate and base composition independent of DAF. Numerical values for *B* and c are shown above each bar. Underlying data can be found in S1 Data.

To validate the results obtained with *M*, we repeated the equilibrium calculations using de novo mutations from parent-offspring trio sequencing. Even though these produced very noisy graphs (due to the paucity of data, S1 Table), they generally resembled the equilibriums calculated by *M* (S6G-S6J Fig).

Differences between mutational equilibria and substitution equilibria indicate that nucleotide levels are being impacted by changes in fixation probabilities due to either selection or gBGC since the species have diverged. Interestingly, the difference between equilibrium GC content by mutations, as predicted by *M*, and substitution rates showed a clear peak just downstream of the TSS (Figs 4E, S6E). This suggests that some evolutionary force, either selection or gBGC, has been resisting the depletion in GC-content since the human-chimpanzee common ancestor. In addition to this peak, there was a much broader constant difference throughout the entire region that is likely due to a baseline level of gBGC throughout the genome, known as background gBGC [64].

Next, we calculated the difference between substitution equilibriums and actual GC levels (excluding CpGs) (Figs 4F, S6F). We observed a prominent peak in the difference near the TSS, with the centre of the peak again being present just downstream of the TSS. This difference means that GC content at the TSS is not at the substitution equilibrium. This observation agrees with our previous study [13] and other findings [15], and indicates that ancient evolutionary trends, restricted to a period before the human and chimpanzee lineages diverged, had elevated GC content above its current substitution equilibrium.

We next wanted to determine whether the increase in the fixation of GC over AT near the TSS (as seen in Figs 4E and S4F) was still ongoing and whether it was due to selection on exonic regions, where increases in GC content promotes mRNA nuclear export [4]. Thus, we extracted the site frequency spectrum (SFS) of rare SNPs from gnomAD in intergenic regions and from where the ΔGC peak was the greatest (Fig 4E, −150 to +350 around the TSS). We further parsed regions downstream of the TSS to exonic and intronic sites, as the former should be under selection while the latter should not. We also examined sites of germline recombination hotspots as a positive control for gBGC. We then calculated the ratio of weak to strong (WS, SNPs towards GC) and strong to weak (SW, SNPs away from GC) alleles. One important note is that a significant number of SNPs in the gnomAD dataset are mispolarized between the ancestral and derived alleles. This polarization is negligible at low frequencies but drastically affects alleles in the higher frequency bins (S3 Table). We therefore polarized the alleles based on ENSEMBL’s ancestral sequence for the human genome [63]. We observed that throughout all derived allele frequency (DAF) bins, the SW/WS ratios around the TSS are similar to that of intergenic regions. When exonic and intronic regions are examined separately they too have the same ratios. This suggests that beyond background gBGC, there is a lack of selection for GC around the TSS. Note that all ratios increase slightly (from ~0.5 at low DAF bins to ~0.6 at high DAF bins), consistent with background gBGC. For recombination hotspots, the ratio starts to deviate from the rest at around DAF bin 0.01 and rises drastically after DAF bin 0.2. This is a clear signal of gBGC, which is not observed for sites around the TSS.

From this analysis we conclude that it is very unlikely that the increase in GC at the start of genes is due to selection, either in exons or at promoters, as we would expect this type of evolutionary force to be ongoing. Instead, the fluctuation in fixation biases towards GC is best explained by punctuated episodes of TSS-associated gBGC. Our data strongly indicates that the lineage that gave rise to the human-chimpanzee ancestor, experienced TSS-associated gBGC episodes (Fig 4F). After humans split off from chimpanzees, our lineage experienced a limited amount of gBGC (Fig 4E). Then, in more recent evolutionary history, we see a lack of TSS-associated gBGC (Fig 4G). Since this last analysis is based in part on high-frequency human variants, where the majority have an estimated age of 40,000–500,000 years [65], it is likely that there have not been any punctuated TSS-associated gBGC episodes over this timescale.

### Estimation of gBGC strength (*B*) at TSS regions

We used the polarized WS and SW polymorphism data above (Fig 4G) to estimate the population-scaled gBGC coefficient *B* for each region. We followed the diffusion-based framework of Glémin, which estimates *B* from the relative distribution of WS and SW polymorphisms across the DAF spectrum [64]. Instead of fitting the absolute DAF, we estimated for each frequency class, the proportion of WS among SW + WS mutations. Because demography affects both WS and SW mutations similarly, much of the demographic distortion cancels in this conditional ratio, making the inference of *B* robust [64].

To separate the contribution of gBGC from regional differences in baseline mutation rates and W versus S base availability, we included a second parameter, *c*, which captures the expected WS:SW count ratio under *B* = 0. This parameter takes into account asymmetries introduced at the mutation stage, and differences in local base composition. Because these processes affect the creation of mutations rather than their subsequent frequency dynamics, they are expected to primarily alter the full WS:SW ratio without producing the DAF-skew generated by gBGC.

Across all regions, the intergenic estimate of *B =* 0.47 (95% CI: 0.46–0.47) reproduced previous estimates of the background gBGC (between 0.3–0.5 [64,66]), confirming the method’s calibration. Recombination hotspots gave *B* = 2.45 (95% CI: 2.40–2.50), within the expected range for human hotspots [64,67] and consistent with the steep DAF-dependent rise visible at these regions (Fig 4G). In contrast, the TSS gave *B* = 0.33 (95% CI: 0.25–0.40), and the exon and intron sub-windows gave *B* = 0.36 and 0.22, respectively. Thus, even when we explicitly model the DAF spectrum to extract gBGC strength, the TSS region shows no excess gBGC relative to genome-wide background. Note that the value of *c* is low for TSS, consistent with high GC content in these regions, providing fewer raw opportunities for WS mutations.

From this analysis we concluded that the TSS is experiencing low levels of background gBGC estimated between 0.25 and 0.40, and there is no evidence of recent (in the last ~500,000 years) TSS-associated hotspot gBGC episodes in modern humans.

### In silico modeling of nucleotide content based on localized mutation rates and gBGC

Our previous analyses (Fig 4A-4H) did not explicitly account for CpG dynamics, despite their potential impact on equilibrium nucleotide content estimates. To incorporate these effects and estimate additional parameters, such as TSS-associated ancestral gBGC, we developed a forward-in-time simulation algorithm. We modeled the evolution of synthetic TSS regions while incorporating different localized inferred mutation rates (including CpG decay) and gBGC. The model randomly assigns 5,000 base pairs surrounding the TSS (from −2,500 to +2,500) based on the average GC-content of the human genome (42%). For each gene, it assigns several features: a nucleotide at the TSS to the midpoint (labeled 0) using the known distribution of human TSS nucleotides [47]; a 5'SS motif to an exon intron boundary using known positional [68] and motif [69] distributions; and a translational start site codon, based on the distribution of 5'UTR sizes from human protein-coding genes [70]. The model then calculates a matrix of mutation rates per position for each gene by starting off with the known genome-wide rate for all 12 mutations and CpGs. By fitting Gaussian curves, we then calculated the decrease in CpG mutations around the TSS using our new estimates (Fig 1D-1E) and incorporated this into our matrix. Finally, we increased the base mutation rate around the TSS by fitting two Gaussian curves to match the two main peaks seen in our new estimate of the overall mutation rate at the TSS (see Fig 1C, red line). This matrix modifies the base mutation rate, which in humans is somewhere between 1.12 × 10^-8^ (Fig 1B, black line) and 1.2 × 10^-8^ [71] mutations per nucleotide. This has been set to 10^−3^ mutations per nucleotide in the simulation, indicating that each round represents about 8 × 10^4^ to 9 × 10^4^ generations (assuming that the mutation rate per generation remains constant).

Several factors will then either increase or decrease the probability that a mutation fixes (i.e., fixation rate) and we incorporated this into our model by adjusting the mutation matrix, transforming it into a fixation matrix. Using estimates from ENCODE, we decreased the fixation rate in the 5'UTRs by 20% and the coding region by 70% (see Fig 11B in [72]). Using our estimates of TSS-associated background gBGC (denoted as *B*_*b*_) (Fig 4H), we set it at 0.5 and used it to adjust all WS and SW mutation rates. We also modeled ancient increases in GC-content at the TSS due to these regions being used as recombination hotspots, as evidenced in our observation of increase gBGC events at the TSS before the human-chimp lineages diverged (Fig 4F). This results in an additional long-term ancestral *B* (denoted as *B*_*add*_) at the TSS due to enhanced recombination. Note that when recombination is happening at the TSS, the overall *B* in this region (denoted as *B*_*TSS*_) is the sum of *B*_*b*_ and *B*_*add*_. We modeled this effect as a Gaussian curve with a maximum *B*_*TSS*_ at 2.25, which is slightly lower than PRDM9-associated hotspots (Fig 4H) [64], but gave good preliminary results. The position of the Gaussian curve maxima was set to be located at 80 nucleotides downstream of the TSS, where GC-content is highest, and its width (or σ) was set at 300 nucleotides. This width will reflect both the average gene conversion tract length (estimated to be 75–123 nucleotides for non-crossover gene conversions, which outnumber crossover gene conversions by an order of magnitude [43,73]) and the variability in the position of the gene conversion tract with respect to the TSS. Finally, we incorporated a time interval for how long ago excess recombination at the TSS was turned off. At this point, *B* becomes uniform across the entire 5kb region (with *B*_*add*_ = 0). Looking at a collection of primates, Joseph and colleagues found strong evidence for TSS-associated gBGC only in *Lemur catta* [15], which diverged from humans approximately 63 MYA [74]. In light of this, we let the simulation run for 500 rounds where there is enhanced recombination at the TSS (where *B*_*add*_ = 1.75, and thus *B*_*TSS*_ = 2.25) and then added 100 rounds of our simulation where there was no extra recombination at the TSS and thus *B*_*add*_ = 0. This last time interval represents about 8 × 10^6^ to 9 × 10^6^ generations. If the average generation time for primates is considered to be 6–10 years, this is equivalent to about 50–90 million years.

Using these parameters, we simulated the evolution of 2,000 genes and plotted their average nucleotide content. The simulation matched the observed trends well in the intergenic regions, but diverged significantly starting just upstream of the TSS and continuing downstream from there (Fig 5A).

**Fig 5:**
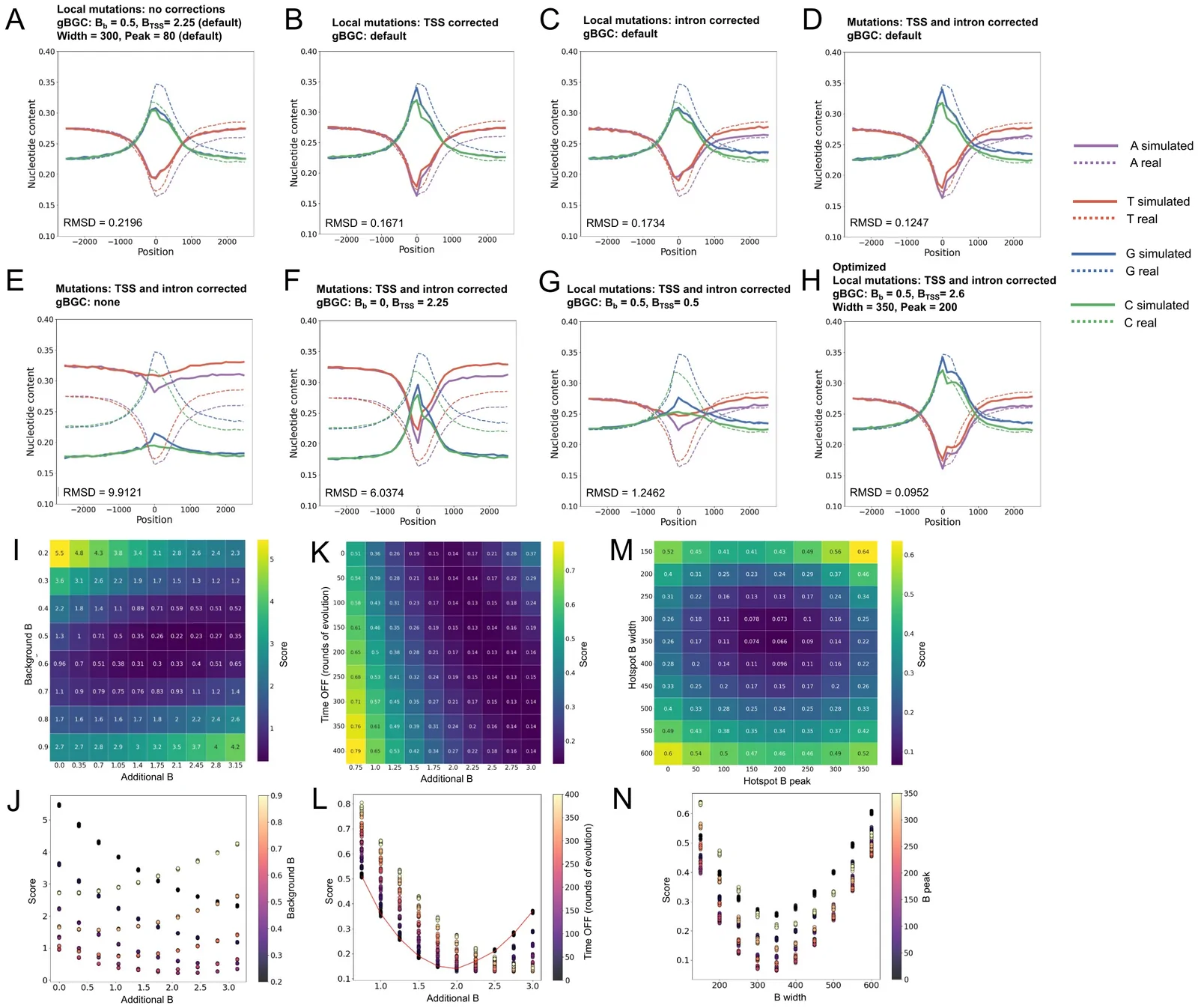
*In silico* simulations of the nucleotide content of human protein-coding genes. **A-H**) Results of *in silico* forward-in-time simulation algorithm to model the evolution of synthetic TSS regions. Solid lines are the average of 2,000 simulated genes. Dotted lines are the average nucleotide content of all human protein-coding genes. Various parameters (local mutation rates around the TSS and/or introns, *B*_*b*_ and *B*_*add*_/*B*_*TSS*_) were added or removed. Optimized parameters are used in (H). Root mean squared deviations (RMSDs) are indicated. Underlying data can be found in S1 Data. **I-J**) Simulations were conducted with various *B*_*b*_ and *B*_*add*_. For each combination, the resulting nucleotide patterns were compared to actual nucleotide values to minimize the RMSDs (scores). Underlying data can be found in S1 Data. **K-L**) Simulations were conducted with various *B*_*add*_ and rounds with *B*_*add*_ = 0 (Time OFF). For each combination, the resulting nucleotide patterns were compared to actual nucleotide values to minimize the RMSDs (scores). Simulations in (L) with Time OFF rounds = 0, are connected by a red line plot. Underlying data can be found in S1 Data. **M-N**) Simulations were conducted with various widths (σ represents one standard deviation of the normalized curve of the TSS-associated gBGC effect) and positions downstream from the TSS that experience additional recombination (“peak” represents the center of the normalized curve of the TSS-associated gBGC effect) where *B*_*TSS*_ is applied. For each combination, the resulting nucleotide patterns were compared to actual nucleotide values to minimize the RMSDs (scores). Underlying data can be found in S1 Data.

Next, we incorporated individual inferred mutation rate corrections for nucleotides near the TSS (see Figs 1F-1Q and S7, black curves). Of all individual inferred mutation rates assessed, G to A (S7D Fig) diverged the most from the overall mutation rate (S7 Fig, bright red curves). We reran the simulation incorporating changes from four additional mutations that diverged the most from the overall rate. After assessing the effect of these four mutations alone or in combination, we determined that simulations that include the G to A residual rate alone (Fig 5B) had the lowest Akaike Information Criterion (AIC) among all models, indicating the best trade-off between model complexity and fit measured by likelihood (see Methods).

We then incorporated inferred mutation rate corrections for nucleotides in the intron (see S3 Fig, “around exon/intron boundary” column 4, and S8 Fig). This time we reran the simulation with seven mutations (alone or in combination) and determined that including three (C to A, T to A and G to C) gave the lowest AIC. Incorporating these three corrections, without (Fig 5C) and with (Fig 5D) the TSS correction not only decreased the root mean squared deviation (RMSD) but generated much of the strand asymmetry seen in the real data.

Next, we wanted to observe how much of an effect gBGC had on our simulations to assess its role in the evolution of TSS content. When all the effects of gBGC were omitted, the simulations were off by a wide margin (Fig 5E). Adding back just *B*_*b*_ or just *B*_*add*_, improved the simulation a bit (Fig 5F-5G), but was not as good as when both were incorporated (Fig 5D).

We then proceeded to optimize *B* (the final simulation with optimizations is shown in Fig 5H). To do so, we reran the simulations with various *B*_*b*_ and *B*_*add*_. We found that values with the lowest RMSD were *B*_*b*_ = 0.5 and *B*_*add*_ = 2.1 (making B_TSS_ = 2.6) (Fig 5I-5J). Next, we wanted to examine the relationship between the strength of long-term gBGC at the TSS and the period of time when there was no extra recombination at the TSS (Time OFF) (Fig 5K-5L). Note that the data points for Time OFF = 0 rounds are highlighted (red line in Fig 5L). This line highlights that Time OFF = 0 produces a global minimum score distribution when *B*_*add*_ < 2, and produces a maximum when *B*_*add*_ > 2. Note that we observed that slight increases in *B*_*add*_ would require very long periods of additional rounds without recombination to match current nucleotide levels. This likely reflects the fact that the decay in GC content that is experienced at the TSS when there is no extra recombination is very gradual. Finally, we reran the simulations varying the width and central position of *B*_*TSS*_ and found that the lowest RMSD scores had values where the width = 350, and the peak position = 200 nucleotides downstream from the TSS (Fig 5M-5N). This was much further downstream than we had anticipated but was closer to our measurements of ancient gBGC (see Fig 4E-4F). When we incorporated the optimized values (*B*_*b*_ = 0.5, *B*_*TSS*_ = 2.6, width (“σ”) of TSS-associated gBGC = 350, center of TSS-associated gBGC = 200, and rounds of no TSS-associated gBGC = 100), this gave a simulation that closely matched the observed values for all human protein-coding genes (Fig 5H).

In summary, our *in silico* modeling suggests that biased mutations and gBGC played important roles in shaping nucleotide content around the TSS of protein-coding genes. The model predicts a *B*_*b*_ value close to 0.5 near the TSS consistent with the estimate obtained from our DAF spectrum analysis (0.25–0.4, Fig 4H). Our model also indicates that *B*_*TSS*_ was centered substantially further downstream than we originally expected, but that its strength likely matched what we currently see at recombination hotspots.

## Discussion

Here we provide evidence that the transcriptional start site of protein-coding genes is a hotspot for inherited de novo mutations and that these contribute to gene evolution. Our data suggest that these mutations are caused in part by paused RNA Polymerase II activity in germline cells (Fig 3A). Some of our data is in line with a recent study [33] reporting excess inherited mutations near human protein-coding TSSs. Interestingly, that study did not detect the same peak in parent-offspring trio sequencing analysis and instead attributes the increase to mutations that occurred in the germline post-zygotically. In our study, we used a larger set of parent-offspring trio sequencing data and much smaller window sizes. It is worth noting that the amplitude of the mutational peak at TSSs in the parent-offspring trio sequencing data is substantially lower than what is predicted by the *M* metric (compare Fig 3C to Fig 3A), and lower than at RNA polymerase II pause sites (Fig 3C). As suggested by Cortés Guzmán et al., the difference in peak size may be due to post-zygotic mutations that are filtered out of de novo mutation analyses from trio-sequencing [33]. Beyond this finding, our analyses suggest that this mutational hotspot, along with other non-adaptive evolutionary processes, most notably gBGC, helps to shape the nucleotide content around the TSS.

It is likely that other transcribed regions are subjected to elevated mutation rates. For example, it has been seen that regions transcribed by RNA Polymerase III are also elevated in inherited de novo mutations [75]. It has also been observed that origins of replication impact the nucleotide content of nearby genes in a distance- and orientation-dependent manner [32, 76–78]. These patterns suggest that differential replication errors and/or repair events accumulate during the replication of leading or lagging strands, a phenomenon known as R-asymmetry [31], and have impacts on nucleotide content of certain genes. It has also been suggested that transcription during spermatogenesis promotes DNA repair in human and mouse genes [79], and this could alter mutation spectra in gene bodies. Others have documented reduced mutation rates in gene bodies in *Arabidopsis thaliana* [80], although these remain controversial [81,82]. Our analysis suggests the reverse in human genes, particularly the region surrounding the TSS.

Our analysis is contingent on the assumption that *M* ~ *μ*, and that low frequency SNPs in these regions have not experienced much selective pressure. If selection were significantly altering the makeup of this pool, it would mostly purge deleterious mutations and thus suppress variation. However, since most of the inferred mutation rates that we document using *M* are significantly higher than the background rate (Fig 1B), this difference cannot be attributed to negative selection. Although these higher rates would be consistent with recent positive selection, this would be highly unlikely as it would require greatly altered SFS for these mutations, with a skew towards higher frequencies, and this is not seen in the data (Fig 2D).

Our data supports the notion that strand asymmetry in the nucleotide content (also known as skew) of genes can be explained by localized changes in mutational rates that are strand-asymmetric (e.g., that G to A and C to T rates are different on a given strand). Our observations and simulations indicate that trends in de novo mutations near the TSS likely contribute to the nucleotide content of the first exons and first introns. These results may explain why introns tend to be T/U-rich and have extreme strand asymmetry [12]. Our findings that mutations differ on coding and template strands may explain some of the strand asymmetry that is found in genes, and mirror previous observations that were based on substitution rates [3].

Our analyses also clarify how current patterns of recombination contribute to the nucleotide content at the TSS. The strength of background gBGC near the TSS (*B*_*b*_ = 0.25 to 0.40, Fig 4H, and *B*_*b*_ = 0.5, Fig 5H) is similar to other genome-wide estimates (between 0.3–0.5 [64,66]). Our estimation of *B*_*b*_ in Fig 4H assumes constant effective population size, no purifying selection on W ↔ S polymorphisms, and stationary mutation signatures across DAF bins, which are standard assumptions in population genetic analyses. Having said this, it is possible that the filtering out of CpGs may lead to slight changes in DAF ratios, thus explaining the slight discrepancy between our two estimates of *B*_*b*_.

Our analyses (Fig 4E-4G) and simulations (Fig 5H, 5I–5N) provide insights into how ancient recombination events shaped the GC-content of human genes. The strength of *B*_*TSS*_, which is a measure of the long-term gBGC strength, depends on how long ago our lineage stopped experiencing elevated recombination around TSSs (Fig 5K-5L). It is likely that these ancient gBGC events were due to both crossover- and non-crossover-catalyzed gene conversion events, although the latter outnumber the former by an order of magnitude. Recent studies have begun to clarify the genome-wide distribution of non-crossover gene conversion events [43,83]. In humans, they tend to correlate with recombination hotspots but are enriched in highly transcribed regions, especially in exons, beyond what is predicted by recombination rates [43]. Despite this, their relation to TSSs in various PRDM9 allele backgrounds (i.e., in species with high levels of TSS-associated gBGC) needs further investigation.

From our simulations, the region affected by *B*_*TSS*_ is centered further downstream than we anticipated (~200 nucleotides, Fig 5M-5N). Indeed, when we compare substitution equilibria and actual GC-content, we can see that the residual, which we argue is due to gene conversion tracts from ancient recombination sites, also gives a peak that is downstream from the TSS (see Fig 4E-4F). The center of this peak may still be dictated by RNA Polymerase II pausing (which occurs 20–60 nucleotides downstream from the TSS), likely when it encounters the first nucleosome [84]. One possible explanation is that transcriptional bubbles generate supercoiling that is relieved by topoisomerases acting downstream from paused RNA Polymerase II. Indeed, topoisomerases induce double-stranded breaks in these regions [26], which mirrors the activity of the topoisomerase, SPO11, which initiates the double-stranded break at PRDM9 sites.

Our models predict that the width of *B*_*TSS*_ = 350 nucleotides (the σ of a gaussian curve, Fig 5M-5N). This peak width is a combination of gene conversion lengths, estimated to be 75–123 nucleotides long [43,73], and the variability in the central position of gene conversion tracts. These likely vary between genes, and between individual events at the same gene. When compared to PRDM9 motifs, the center of non-crossover conversion tracts tends to fall within a 50–100 nucleotide window (see S6 Fig in [43]). If we assume a similar variability in the center of the tracts, this combined with tract length may explain our overall width.

Our study highlights the importance of non-adaptive evolutionary forces in shaping the nucleotide content at the 5'end of genes. The non-adaptive sculpting of genomic features may represent an important mechanism underlying the emergence and the maintenance of genomic landmarks in organisms that evolve under weak selection regimes, such as humans. In the absence of these non-adaptive forces, selection alone may be insufficient to maintain many of these landmarks. Instead, selection may primarily act on molecular processes, such as those involved in the mRNA metabolism, to recognize and use these genomic patterns that are largely influenced by non-adaptive evolutionary forces.

## Methods

### Sequence data and annotation

Sequences of Human (*Homo sapiens*, GRCh38, release 108), Chimpanzee (*Pan troglodytes*, Pan_tro_3.0, release 108), and Gorilla (*Gorilla gorilla*, gorGor4, release 108) genomes were accessed and downloaded from the Ensembl database (www.ensembl.org). Annotations for protein-coding genes were retrieved using the University of California Santa Cruz (UCSC) Genome Browser annotation track database based on the corresponding genome assemblies. For mapping rare SNPs and de novo mutations, annotations for the TSS were obtained from testis tissue-specific Cap Analysis of Gene Expression (CAGE) sequencing from the FANTOM5 project [36]. The best TSS from CAGE-seq was defined as the transcript with the highest tags per million score. This set of testes-specific CAGE-seq annotated genes was used as our default set of “protein-coding genes” unless stated otherwise. For mapping substitutions between species, positions for the TSS were determined by the most commonly used start site of all transcripts per gene based on ENSEMBL annotation as described previously [13]. The exon-intron boundaries corresponding to the TSSs were obtained from Gene Transfer Format annotation files from Ensembl (https://ftp.ensembl.org/pub/release-108/gtf/homo_sapiens/)

Human lncRNAs, with accurate 5’ends, were obtained from FANTOM CAT [85]. Testis samples were identified within the FANTOM-CAT gene-level expression atlas by cross-referencing sample library IDs (CNhs codes) against the FANTOM5 human tissue sample metadata file (HumanSamples2.0 SDRF), retaining columns whose sample description contained the keyword “testis.” For lncRNAs not expressed in testis, genes with Counts Per Million of over 0 in any of the three adult testis samples were excluded.

RNA Polymerase II pause sites were obtained from ENCODE ChIP-seq (https://www.encodeproject.org/experiments/ENCSR803FAP/; GEO:GSE105816), and filtered for pause sites in intergenic regions and greater than 1kb from other TSSs. We used the middle of the annotated ChIP-seq coverage to align each site.

To filter for genes not expressed in testes, we used gene annotations from ENSEMBL as described above instead of CAGE-seq. Tissue-specific RNA transcript expression was obtained from the Human Protein Atlas (https://www.proteinatlas.org/humanproteome/tissue/data#consensus_tissues_rna) [86], and only protein-coding genes with transcript per million (TPM) of 0 were included.

Intergenic genomic regions were obtained by randomly generating genome coordinates outside of protein-coding regions. (Github: https://github.com/tinaqiu221/GC_evolution/generate_random_coordinates.py).

Recombination hotspots were identified by intersecting two complementary genome-wide datasets. Meiotic double-strand break (DSB) hotspot coordinates were obtained from [87], who mapped DSB initiation sites genome-wide in human testis tissue using DMC1 chromatin immunoprecipitation followed by single-stranded DNA sequencing (SSDS). Peak coordinates for PRDM9-A homozygous individuals (AA1 and AA2), representing the most common PRDM9 allele in European-ancestry populations, were downloaded from the Gene Expression Omnibus (GEO: GSE59836; GSE59836_Peak_data_Supplementary_File_1.txt.gz). Hotspot coordinates were originally mapped to hg19 and lifted over to GRCh38 using the UCSC liftOver utility with the hg19ToHg38 chain file (https://hgdownload.soe.ucsc.edu/goldenPath/hg19/liftOver/). Crossover rate data were retrieved from the UCSC Genome Browser recombAvg track via the UCSC REST API (https://api.genome.ucsc.edu), and regions with a recombination rate of ≥50 cM/Mb were retained as crossover hotspots, corresponding to approximately 40-fold above the genome-wide average. Intervals narrower than 500 bp were excluded as bigWig boundary artifacts. Final high-confidence germline recombination hotspots were defined as the intersection of DMC1-SSDS DSB peaks with crossover hotspot intervals using BEDTools intersect [88]. (Github: https://github.com/tinaqiu221/TSS_hypermutation/recombination_hotspots_pipeline.sh).

Nucleotide sequences from all desired regions were retrieved using the BEDTools *getfasta* suite (https://bedtools.readthedocs.io/en/latest/content/tools/getfasta.html)

### Rare SNP mapping and derivation of M

Human single nucleotide polymorphisms (SNPs) were obtained from the gnomAD v3.0 database, which contains 71,702 whole genome sequences across 9 population groups mapped to the GRCh38 genome build (https://gnomad.broadinstitute.org/news/2019-10-gnomad-v3-0/). SNPs with a frequency of less than 1% (for *M*_*1%*_) or less than 0.1% (for *M*_*0.1%*_) in all subpopulation groups were filtered using Hail python package (https://pypi.org/project/hail/). Rare SNPs were mapped 1kb around TSSs of protein-coding genes, from the front of exon 1, from the back of exon 1, around first exon-intron boundaries, around TSSs of lncRNAs expressed in testes, around TSSs of lncRNAs not expressed in testes, around intergenic RNA Polymerase II pause sites, around TSSs of protein-coding genes not expressed in testes, and around random intergenic regions. The summary mutation table reports the counts of the 12 mutation types, with each count corresponding to the sum of occurrences within a 100 bp window upstream (−400 to −300 relative position), around TSS or focal site (−50 to +50 relative position), and downstream (+300 to +400 relative position).

The fraction of nucleotides with SNP frequency below the cutoff *x* (*F*_*x*_) was calculated by dividing the total count of rare SNPs per position by twice the number of nucleotides analyzed per relative position (to account for diploidy). In most cases, all mutations were considered; however to calculate *B* (i.e., Figs S5 and 5), CpG mutations were filtered out by excluding CpG > TpG and CpG > CpA mutations from the numerator and CpG positions from the denominator for C to T and G to A mutation types. To account for the fact that multiple mutations at the same site are independent and can lead to underestimation of the true rare SNP fraction (*F*^*t*^), we transformed *F*:


$$\begin{array}{c} F_{x}^{t}=-\text{ln}(\text{1}-\;F_{x}) \end{array}$$

*F*^*t*^ and genome sample number (*N*) were used to obtain the metric *D*_*x*_ (population-scaled SNP density per site, for bin *x*):


$$\begin{array}{c} D_{x}=\frac{F_{x}^{t}}{N} \end{array}$$

*D*_*x*_ can be interpreted as the probability that a position contains a recently created de novo mutation (i.e., a rare SNP less than frequency *x*) that is observable in one member of the population. At equilibrium, and assuming limited temporal changes in parameters (*N*_*e*_, *s*, and *b*), the number of SNPs gained in a region of length *L* (that are in bin 0 to *x* frequency) in the surveyed population (*N*) must equal the number of SNPs in this same region that are lost from the bin in this population. Assuming that the number of SNPs gained due to de novo mutations in a generation is vastly greater than the number of higher frequency SNPs that drop below *x* frequency, this gives:


$$\begin{array}{c} \mu LN=\frac{F_{x}^{t}L}{G_{x}} \end{array}$$

which simplifies to:


$$\begin{array}{c} \mu =\frac{D_{x}}{G_{x}} \end{array}$$

In this equation, *G*_*x*_ is the mean number of generations a SNP is present in the ascribed bin (from 0 to *x* frequency) before being lost. It also acts as a rescaling factor between *μ* and *D*_*x*_. Note that *G* will depend on local *N*_*e*_, *s* and *b*. Using *μ* and *D* values for intergenic regions, we can estimate *G* intergenic (*G*^*I*^) to be *G*^*I*^_*1%*_ = 105 generations and *G*^*I*^_*0.1%*_ = 97 generations. Also note that our calculated intergenic mutation rate (1.12x10^-8^ mutations/base) is equivalent to 74 mutations per generation per diploid genome.

For regions such as the TSS, we can use local values of *D* (in this case *D*_*x*_^*T*^) to obtain a metric *M*_*x*_^*T*^:


$$\begin{array}{c} M_{x}^{T}=\frac{D_{x}^{T}}{G_{x}^{I}} \end{array}$$

Assuming that *G* is the same in intergenic and TSS regions (i.e., that *G*^*I*^ ≈ *G*^*T*^), which is true when *s* and *b* are small and local *N*_*e*_ in these two regions are similar, then *M*^*T*^ ≈ μ. Unless stated otherwise, *M* refers to *M*_*1%*_.

In calculations of the fractional change between *M*_*1%*_ and *M*_*0.1%*_, CpG mutations were omitted as these significantly contribute additional SW mutations that exaggerate the fractional change due to gBGC effects (an increase in SW mutations to *M*_*1%*_ and depletion to *M*_*0.1%*_ as can be seen in S8 Fig).

All codes were compiled and annotated in Jupyter Notebook (Github: https://github.com/tinaqiu221/TSS_hypermutation/RareSNP.ipynb).

### 5-mer sequence context rate calculation

Overall baseline mutation rates for each nucleotide were first calculated using rare SNPs mapped to random intergenic regions. For each nucleotide, mutation and background counts were pooled across all 5-mer contexts sharing that centre base, and the pooled proportion was converted to a rate via *F*^*t*^ = −ln(1 − *F*). The intergenic sequences were then scanned to count all possible 5-mer contexts (the centre base flanked by two upstream and two downstream nucleotides), providing background context frequencies. For each 5-mer, a context-specific change value was computed as the context mutation rate divided by baseline rate for that nucleotide, representing the relative mutability of each context compared to the genome-wide average. The same procedure was applied to rare SNPs mapped to 1kb around regions of interest (TSSs of protein-coding genes, TSSs of lncRNAs expressed in testes, TSSs of lncRNAs not expressed in testes, intergenic RNA Polymerase II pause sites, TSSs of protein-coding genes not expressed in testes). Mutations were assigned to positions relative to the feature anchor (e.g., TSS), and a 1,024 × 1,001 count matrix was constructed (5-mer contexts × positions). Each count was weighted by its corresponding change value. At each position, the weighted sum was divided by the total raw count to produce a context-corrected rate. This rate was multiplied by the intergenic base mutation rate to give the absolute expected mutation rate per position, reflecting the contribution of local sequence composition to mutational patterns across the region. 5-mer context rates were also calculated excluding positions and mutations in CpG contexts.

All codes were compiled and annotated in Jupyter Notebook (Github: https://github.com/tinaqiu221/TSS_hypermutation/5mer_context_rates.ipynb).

### Single generation de novo mutation mapping

Human de novo mutations from parent-offspring trios were compiled from seven different sources (S2 Table) [37–43], removing overlaps. Mutations were normalized by the number of contributing trios to obtain per-generation estimates, and mapped to 1kb regions around either TSSs of protein-coding genes, TSSs of lncRNAs, intergenic RNA Polymerase II pause sites, TSSs of protein-coding genes not expressed in testes, and random intergenic regions. Summary mutations per region and mutation rates were calculated similarly to the above rare SNPs mapping. All codes were compiled and annotated in Jupyter Notebook (Github: https://github.com/tinaqiu221/TSS_hypermutation/DNM.ipynb).

### Somatic mutation mapping

Somatic mutations from normal human tissues were obtained from SomaMutDB 2.0, which aggregates whole-genome sequencing data from 47 published studies encompassing 30 tissue types [89]. Nine datasets involving tissues with known DNA repair deficiencies, disease conditions, or exogenous mutagen exposure (including Cockayne syndrome and xeroderma pigmentosum neurons, ulcerative colitis and IBD-affected colon, cirrhotic liver, BRCA1/2 carriers, and treatment-exposed samples) were excluded, leaving mutations from healthy tissues across 38 studies. Somatic mutations were mapped 1kb around the TSS of protein-coding genes. Outlier loci contributing disproportionate mutations at individual positions were identified and excluded. All codes were compiled and annotated in Jupyter Notebook (Github: https://github.com/tinaqiu221/TSS_hypermutation/Somatic_mutations.ipynb).

### Nucleotide substitution mapping

Substitutions for human and chimpanzee using gorilla as an outgroup were obtained as previously described [13] (Github: https://github.com/tinaqiu221/GC_evolution/get_homologous_sequences.py.ipynb, https://github.com/tinaqiu221/GC_evolution/needleman_alignment.py). Substitutions were also performed using ENSEMBL EPO primate inferred ancestral sequence [63]. (Github: https://github.com/tinaqiu221/TSS_hypermutation/ancestral_substitution.ipynb).

### Equilibrium nucleotide content calculation

Predicted equilibrium nucleotide frequencies were computed from rare SNP *M*_*1%*_, de novo mutation rates, or substitution rates, divided into either 100 bins (50 positions per bin for 5kb around the TSS), or 40 bins (25 positions per bin for 1kb around the TSS). All rates are calculated by dividing mutational count by mutational opportunities. For each genomic bin, a 4x4 rate matrix Q was constructed, with diagonal elements set so rows summed to zero. The equilibrium distribution π was obtained by solving πQ = 0 using least-squares optimization, subject to Σπ = 1. Predicted equilibrium frequencies were compared against actual nucleotide compositions across 100 bins or 40 bins spanning 5kb or 1kb positions around the TSS. Mutations in a CpG context were parsed out. GC difference was calculated either by subtracting the average of human and chimp predicted equilibrium GC based substitutions rates by the predicted equilibrium GC content based on *M*_*1%*_, or by subtracting the actual GC content by the average of human and chimp predicted equilibrium GC based on substitutions rates. GC differences use a sliding window of 250 nucleotides.

All codes were compiled and annotated in Jupyter Notebook (Github: https://github.com/tinaqiu221/TSS_hypermutation/Equilibrium.ipynb).

### Site frequency spectrum analyses

Single-nucleotide variants and global allele frequencies were extracted from the gnomAD v3.1.1 sites Hail Table for three genomic region sets: a −150 to +350 bp around the TSS of protein-coding genes, deCODE-defined recombination hotspots, and a control set of random intergenic regions. Variants downstream of the TSS window were further classified as exonic or intronic using GENCODE v40 exon coordinates.

Alleles were polarized using the ENSEMBL EPO primate inferred ancestral sequence [63], restricting to high-confidence ancestral calls. For each variant, the derived allele frequency (DAF) was computed as the alt-AF when the reference matched the ancestor, or 1 − alt-AF when the alternate matched the ancestor. Variants with no ancestral call or with an ancestor matching neither allele (recurrent/triallelic) were excluded. Polarized SNPs were classified as weak-to-strong (WS: A to C, A to G, T to C, T to G) or strong-to-weak (SW: G to A, G to T, C to A, C to T) substitutions and binned by DAF.

To account for differences in mutational opportunity across regions of differing GC content, the WS/SW ratio in each DAF bin was multiplied by the regional ratio of strong (G + C) to weak (A + T) base counts (n_S/n_W). The pipeline outputs, for each region, the GC-normalized WS/SW ratio across log-spaced DAF bins from 10^−4^ to 1.

All codes were compiled and annotated in Jupyter Notebook (Github: https://github.com/tinaqiu221/TSS_hypermutation/SFS.ipynb).

### Estimation of B

For each region, we estimated the population-scaled gBGC coefficient $B=\text{4}bN_{e}$, where *N_e_* is the effective population size and *b* is the per-generation conversion bias toward strong (G/C) alleles. We followed the Wright-Fisher diffusion-based framework of Glémin et al. [64], in which the derived allele frequency (DAF) distribution under gBGC strength *B* follows

$f(x|B)\propto \frac{\text{1}-e^{-B(\text{1}-x)}}{x(\text{1}-x)(\text{1}-e^{-B})}$

for WS variants, with *B* replaced by *-B* for SW variants.

To minimize the impact of human demography which strongly distorts the SFS due to recent population expansion [90], we did not fit *B* from the absolute SFS. Instead, we estimated the WS proportion among WS + SW for each DAF class. This proportion depends only on *B* because demography affects WS and SW spectra symmetrically and cancels out in the ratio [64]:

$Pws(x|B,c)=\frac{c\cdot g(x;+B)}{c\cdot g(x;+B)+g(x;-B)},g(x;B)=\frac{\text{1}-e^{-B(\text{1}-x)}}{\text{1}-e^{-B}}$

The auxiliary parameter *c* is a region-specific baseline that captures the expected raw WS:SW count ratio under *B* = 0, encompassing per-base mutation rates, W versus S base availability, and any region-specific mutation-rate effects.

For each region, polarized W ↔ S polymorphisms were binned into 30 log-spaced DAF intervals over [10^−4^, 0.95], excluding the rare extreme to avoid singleton/sequencing noise and the fixed-allele pile-up that violates infinite-sites assumptions. Within each bin *i*, observed counts were modeled as Binomial. The joint maximum-likelihood estimate of (*B*, *c*) was found by Nelder-Mead optimization, with 95% confidence intervals on *B* obtained from profile likelihood (cutoff Δ log L = 1.92, corresponding to χ²1 at α = 0.05).

All codes were compiled and annotated in Jupyter Notebook (Github: https://github.com/tinaqiu221/TSS_hypermutation/B_estimation.ipynb).

### In silico modeling of nucleotide content

We developed a forward-in-time nucleotide evolution simulator to model mutation patterns around human TSS. We simulated a sequence of 5kb centered around TSS that mimics human genes that have exons and introns. We thus estimated the size of the first exon of our simulated sequences from the distribution of observed exon lengths. For each set of simulation parameters we simulated 2000 genes. We performed 10 replicates per set of parameters. We integrated 43 parameters from empirical mutation data deduced from gnomAD, including baseline mutation rates, position-specific CpG effects, and regional constraints around exons, introns, and UTRs. We also incorporated GC-biased gene conversion, with both a uniform background intensity and a localized hotspot effect, as well as a phase in which hotspot activity ceases. We evaluated models with and without a position-dependent mutation spectrum around the TSS. Parameter values were optimized to reproduce the observed per-position nucleotide frequencies, and model selection was guided by likelihood-based criteria. Using this framework, we show that both an ancestral hotspot *B* and a mutation spectrum varying along the TSS are necessary to reproduce the empirical GC content patterns. Details of the simulator, parameterization, and fitting procedure are provided in S1 Text.

(Github: https://github.com/tinaqiu221/TSS_hypermutation/Nucleotide_evolution_simulator/).

### COSMIC SBS mutational signatures

Mutational signature activities were analyzed across the 1 kb region surrounding each genomic feature in 25 bp windows, yielding 40 windows per region. For each window, the observed 96 mutation spectrum was generated from called single base substitutions using SigProfilerMatrixGenerator. To correct for differences in trinucleotide composition between each window and the reference genome, trinucleotide opportunities (the number of sites corresponding to each of the 96 contexts) were counted directly from the GRCh38 reference sequence, separately for each local window and for the genome as a whole. Observed mutation counts in each channel were rescaled by the ratio of genome-wide to local trinucleotide opportunity (each expressed as a fraction of its channel total), placing the local spectrum on the same trinucleotide background against which the COSMIC reference signatures are defined. The opportunity-adjusted spectrum was then fit against unmodified COSMIC v3.5 reference signatures (GRCh38 genome build) using non-negative least squares (NNLS) implemented in scipy. Signature activities are reported as the percentage of total mutations attributed to each signature per window. All analyses were performed in Python and are fully annotated in the accompanying Jupyter notebook.

(Github: https://github.com/tinaqiu221/TSS_hypermutation/COSMIC_SBS.ipynb).

### Statistical analyses

To statistically test whether *M* or de novo mutation rate at one locus of a sequence was significantly different from *M* at another locus of the same sequence (e.g., between position −400 and −300, and position −50 and +50 relative to the TSS), the Wilcoxon signed-rank test was performed between the distributions of the 2 loci (Github: https://github.com/tinaqiu221/TSS_hypermutation/Stats_test.ipynb-Wilcoxon).

To statistically test whether the number of net changes in rare SNPs or DNMs at relative nucleotide position 0 was significantly different between two datasets (e.g., between the TSS and random intergenic position), a permutation test was performed [91]. In this test, net changes in rare SNPs or DNMs from datasets A and B were randomly shuffled into two groups 1,000 times, and the differences between the two random groups from the 1,000 randomizations were used to generate a normalized curve. The actual observed delta between datasets A and group B was plotted onto the normalized curve, and the p-value was the area under the curve separated by the line x = observed delta (Github: https://github.com/tinaqiu221/TSS_hypermutation/Stats_test.ipynb – Permutation test). To represent this data in the figures, the normalized curve was plotted as a whisker-bar graph and the observed delta was plotted as a black dot on the same graph.

## Supporting information

S1 Text Simulating the evolution around TSS. (PDF)

S1 Fig Mutation patterns around TSSs of human protein-coding genes. **A**) Single nucleotide resolution of *M*_*1%*_ for ΔGC (change in GC/all countable nucleotides) around the TSS of all human protein-coding genes. Change in GC is all gain of GC mutations minus all loss of GC mutations. Underlying data can be found in S1 Data. **B**) Single nucleotide resolution of *M*_*1%*_ for all mutations including CpGs, over all TSSs from human protein-coding genes (red) or an equivalent number of intergenic regions (black) using low frequency SNP analysis from gnomAD. Single nucleotide predicted rate based on 5-mer sequence context over all TSSs from human protein-coding genes (pink). Underlying data can be found in S1 Data (for 5-mer context). **C-D**) Single nucleotide resolution of *M*_*1%*_, for CpG to TpG (**C**) and CpG to CpA (**D**) around the TSS of all human protein-coding genes. Underlying data can be found in S1 Data. **E-F**) Somatic mutations around the TSS of protein-coding genes based on the SomaMutDB excluding CpG mutations (**E**) and including CpG mutations (**F**). Underlying data can be found in S1 Data.
(TIF)

S2 Fig Single base substitution mutational signature activities across genomic regions. **A**) SBS mutational signature activities across 25 bp windows spanning the 1 kb of various genomic regions, expressed as percentage of total mutations per window, normalized for genome-wide and local trinucleotide opportunity (see Methods). Underlying data can be found in S1 Data. **B**) Pairwise cosine similarity between COSMIC v3.5 SBS reference profiles for all signatures identified as active in (**A**). Underlying data can be found in S1 Data.
(TIF)

S3 Fig Single nucleotide resolution *M* for the first exon of human protein-coding genes. **A-L**) Single nucleotide resolution of *M*_*1%*_, for each indicated mutation type around the TSS (column 1) and first exon-intron boundary (column 4) of all human protein-coding genes. Column 2 is all first exons aligned so that the first nucleotides are positioned at 0. Column 3 is all first exons aligned so that the last nucleotides (at the exon-intron boundary) are positioned at 0. Underlying data can be found in S1 Data.
(TIF)

S4 Fig Mutation rate analyses across genomic regions. **A-B**) Mutation rates based on de novo mutations as inferred by parent-offspring trio sequence analysis for all mutations excluding CpGs, over all TSSs from human protein-coding genes (**A**), and intergenic RNAPII pause sites (**B**). Single nucleotide predicted rate based on 5-mer sequence context over regions indicated are in lighter shaded lines. Rates are plotted as a sliding window of 100 nucleotides. Underlying data can be found in S1 Data. **C**) Single nucleotide resolution of *M*_*1%*_ for all mutations excluding CpGs, over various indicated regions from the human genome. Underlying data can be found in S1 Data. **D-H**) Single nucleotide resolution of *M*_*1%*_ for all mutations excluding CpGs, over all TSSs from human protein-coding genes (**D**), protein-coding genes not expressed in testes (**E**), lncRNA expressed in testes (**F**), lncRNA not expressed in testes (**G**), and intergenic RNAPII pause sites (**H**), using low frequency SNP analysis from gnomAD. Single nucleotide predicted rate based on 5-mer sequence context over regions indicated are in lighter shaded lines. Underlying data can be found in S1 Data.
(TIF)

S5 Fig *M* of individual mutation types in various genomic regions. **A-M**) *M*_*1%*_ over 100 bp windows as indicated in (M) for various indicated regions from the human genome. Statistical significance (p < 0.05) results from Wilcoxon signed-rank tests are indicated in symbols. Underlying data can be found in S1 Data.
(TIF)

S6 Fig Calculated equilibrium levels of nucleotides inferred from *M* and de novo mutation rates based on derived ancestry. **A-D**) The observed (black lines) nucleotide content and predicted equilibrium nucleotide levels based on either *M*_*1%*_ (pink lines) or substitution rates inferred from ENSEMBL’s primate ancestral sequence for human or chimpanzee (blue lines), for regions surrounding the TSS of human protein-coding genes. All plots use bins of 50 nucleotides. Underlying data can be found in S1 Data. **E**) Difference in GC-content between predicted equilibrium levels based on ENSEMBL-derived substitution rates (averaged between human and chimpanzee) and *M*_*1%*_ for regions surrounding the TSS of human protein-coding genes. The values use a sliding window of 250 base pairs. Note that the difference is uniform across the region, suggesting that background gBGC is acting throughout this region. Underlying data can be found in S1 Data. **F**) Difference in GC-content between current nucleotide content in the human genome and predicted equilibrium levels based on ENSEMBL-derived substitution rates (averaged between human and chimpanzee rates) for regions surrounding the TSS of human protein-coding genes. The values use a sliding window of 250 base pairs. Note that the difference is most pronounced in a peak centered just downstream of the TSS of the graph, suggesting that TSS-associated gBGC occurred before the divergence of humans and chimpanzees. Underlying data can be found in S1 Data. **G-J**) The observed (black lines) nucleotide content and predicted equilibrium nucleotide levels based on either *M*_*1%*_ (purple lines) or de novo mutations (green) as inferred from parent-offspring trio sequencing. Observed nucleotide content and equilibrium predicted based on *M*_*1%*_ are in bins of 25 nucleotides, and equilibrium predicted based on both de novo mutations are in sliding window of 125 nucleotides. Underlying data can be found in S1 Data.
(TIF)

S7 Fig Comparison of overall *M* at TSS with individual rates. **A-L**) Normalized inferred mutation rates *M* (grey lines) are compared to line fitted overall inferred mutation rate *M* used in the *in silico* time-forward simulation of nucleotide content evolution (smooth bright red line). The resulting residual (burgundy line) is also plotted. After AIC analysis, it was determined that incorporating the G to A residual (**D**) significantly improved the simulation performance and the resulting mutation rate (fitted overall mutation rate + fitted residual) is also plotted (smooth green line) in (**D**). Underlying data can be found in S1 Data.
(TIF)

S8 Fig Comparison of overall *M* in first introns with individual rates. **A-L**) Normalized inferred mutation rates *M* (grey lines) are compared to line fitted overall inferred mutation rate *M* used in the *in silico* time-forward simulation of nucleotide content evolution (smooth bright red line) with the *x-axes* depicting the distance from the exon-intron boundary (EIB). The resulting residual (burgundy line) is also plotted. After AIC analysis, it was determined that incorporating **C** to **A**, **T** to **A** and **G** to **C** residuals significantly improved the simulation performance. Underlying data can be found in S1 Data.
(TIF)

S1 Table Number of mutations/SNPs per dataset. (DOCX)

S2 Table DNM sources, number of trios and mutations. (DOCX)

S3 Table Mispolarization rate of gnomAD reference/alternate alleles across derived allele frequency bins for each analyzed genomic region. (DOCX)

S1 Data Fig1._Data.xlsx: Underlying data for Figs 1 and S1, including single nucleotide resolution of nucleotide content and *M_1%_* (total, nonCpG, CpG to TpG, CpG to CpA, and individual mutation types) around human protein-coding gene TSSs and intergenic regions, as well as ΔGC, *M_0.1%_*, and somatic mutation data. **Fig2._Data.xlsx:** Underlying data for Fig 2, including single nucleotide resolution of *M_1%_* and *M_0.1%_* around TSSs and first exon-intron boundaries of human protein-coding genes, fractional change between *M_1%_* and *M_0.1%_*,and *M_1%_* around aligned TSSs and first exon/intron boundaries. **Fig3._Data.xlsx:** Underlying data for Figs 3 and S4, including *M_1%_* over various genomic regions, de novo mutation rates from parent-offspring trio analysis, 5-mer sequence context predictions, and mutation rate analyses across genomic regions. **Fig4A-F_Data.xlsx:** Underlying data for Fig 4A-4F, including observed nucleotide content, predicted equilibrium nucleotide levels based on *M_1%_* and substitution rates from human-chimpanzee-gorilla trio analysis, and GC-content differences for regions surrounding human protein-coding gene TSSs. **Fig4G-H_Data.xlsx:** Underlying data for Fig 4G-4H, including weak-to-strong (WS) over strong-to-weak (SW) variant ratios across derived allele frequency (DAF) bins for intergenic regions, recombination hotspots, and TSS windows, and maximum-likelihood estimates of the population-scaled gBGC coefficient B with confidence intervals. **Fig5A-H_Data.xlsx:** Underlying data for Fig 5A–5H, including average nucleotide content (A, C, G, T proportions) at single-position resolution around human protein-coding gene TSSs from in silico forward-in-time simulations (2,000 simulated genes per condition) across progressively refined parameter sets incorporating local mutation rates, *B_b_*, and *B_add_*/*B_TSS_*. **Fig5I-N_Data.xlsx:** Underlying data for Fig 5I–5N, including raw replicate and mean RMSD scores from parameter sweeps of *B_b_* and *B_add_* (panels I–J), *B_add_* and Time OFF rounds (panels K–L), and TSS-associated gBGC effect width (σ) and peak position (panels M–N), used to identify optimal simulation parameters by minimizing deviation from actual human nucleotide content. **FigS2_Data.xlsx:** Underlying data for S2 Fig, including SBS mutational signature activities across 25 bp windows spanning various genomic regions. **FigS3_Data.xlsx:** Underlying data for S3 Fig, including single nucleotide resolution of *M_1%_* for individual mutation types around TSSs, first exons aligned by start or end, and first exon-intron boundaries of human protein-coding genes. **FigS5_Data.xlsx:** Underlying data for S5 Fig, including *M_1%_* over 100 bp windows for individual mutation types across various genomic regions. **FigS6A-F_Data.xlsx:** Underlying data for S6A-S6F Fig, including observed nucleotide content and predicted equilibrium nucleotide levels based on *M_1%_* or ENSEMBL-derived substitution rates, and GC-content differences for regions surrounding human protein-coding gene TSSs. **FigS6G-J_Data.xlsx:** Underlying data for S6G-S6J Fig, including observed nucleotide content and predicted equilibrium nucleotide levels based on *M_1%_* or de novo mutations from parent-offspring trio sequencing. **FigS7_Data.xlsx:** Underlying data for S7 Fig, including normalized residuals between individual inferred mutation rates M and the smoothed overall M for all 12 substitution types at single-nucleotide resolution around human protein-coding gene TSSs, as well as polynomial fit coefficients and AIC-based selection of residual corrections incorporated into the simulation (G to A residual). **FigS8_Data.xlsx:** Underlying data for S8 Fig, including normalized residuals between individual inferred mutation rates M and the smoothed overall M for all 12 substitution types at single-nucleotide resolution around first exon-intron boundaries, as well as polynomial fit coefficients and AIC-based selection of residual corrections incorporated into the simulation (C to A, T to A, and G to C residuals).
(ZIP)

## Abbreviations

5'SS: 5' Splice Sites
AIC: Akaike Information Criterion
CAGE: Cap Analysis Gene Expression
DAF: derived allele frequency
DSB: double-strand break
EIB: exon-intron boundary
gBGC: GC-biased gene conversion
IFB: Institut Français de Bioinformatique
NHEJ: non-homologous end joining
NNLS: non-negative least squares
RMSD: root mean squared deviation
SFS: site frequency spectrum
SNPs: single nucleotide polymorphisms
SSDS: single-stranded DNA sequencing
TMEJ: theta-mediated end joining
TPM: transcript per million
TSSs: transcription start sites
UCSC: University of California Santa Cruz
