# Supplementary material for: Non-adaptive evolutionary forces predominantly shape nucleotide composition near human transcription start sites
Source: PLoS Biol. 2026 Oct 21;24(10):e3004063. doi: 10.1371/journal.pbio.3004063 (PMC13712754; doi:10.1371/journal.pbio.3004063)
Supplement: Supplementary file 1 [file pbio.3004063.s001.pdf]

Simulating the evolution around TSS

To evaluate whether GC-biased gene conversion (gBGC) is required to reproduce the patterns of mutation rate variation and GC content observed around human transcription start sites (TSS), we developed a forward-in-time simulator of nucleotide evolution. The simulator reproduces position-dependent mutation processes inferred from empirical data while integrating all known non-adaptive and adaptive effects that significantly shape local nucleotide composition.

We model mutation rate variation along a 5 kb window centered on the TSS. The model includes (i) baseline mutation processes, (ii) CpG context effects, (iii) fixation bias due to gBGC, and (iv) regional adaptive constraints associated with exonic, intronic, and untranslated regions (UTRs). The goal is to test whether gBGC must be invoked, in addition to these context and constraint effects, to account for the empirical profiles of mutation rates and GC content around TSS.

Baseline mutation process

The empirical baseline mutation spectrum was derived from the human mutation profiles reported in the main paper, based on rare SNPs from the gnomAD dataset. To capture the general mutational background, we used intergenic regions depleted of CpG sites. CpG sites were excluded because they exhibit a distinct mutation dynamic associated with cytosine methylation and deamination, which is treated separately below.

For each mutation type $N_1 > N_2$, with $N_1, N_2 \in \{A, C, G, T\}$, the observed fractions $O_{N_1N_2}$ represent the density of mutations from base N_1 that were observed to change into N_2. Thus, $O_{N_1N_2}$ is not an absolute mutation rate but an observed mutation spectrum normalized per ancestral base.

The genome-wide fractions of mutations originating from AT versus GC bases were defined as:

$$\mu_{\mathrm{AT}} = \frac{O_{AN} + O_{TN}}{O_{AN} + O_{CN} + O_{GN} + O_{TN}}, \qquad \mu_{\mathrm{GC}} = 1 - \mu_{\mathrm{AT}},$$

where $O_{AN} = \sum_{X\in\{C,G,T\}} O_{A>X}$ and similarly for other ancestral bases.

To respect strand symmetry, complementary mutations were pooled (e.g. $A > C$ with $T > G$) and assigned normalized weights:

$$w_{A>X} = \frac{O_{A>X} + O_{T>X^c}}{O_{AN} + O_{TN}}, \qquad w_{G>Y} = \frac{O_{G>Y} + O_{C>Y^c}}{O_{GN} + O_{CN}},$$

with X^c and Y^c denoting complementary nucleotides. The resulting baseline mutation proportions were:

$$\mu_{A>X} = \mu_{\mathrm{AT}} \times w_{A>X}, \qquad \mu_{G>Y} = \mu_{\mathrm{GC}} \times w_{G>Y}.$$

These values define a genome-wide empirical mutation fraction spectrum (comprising twelve mutation types), representing the relative probability of each change before accounting for regional effects, whose specific dynamics are discussed below. The baseline mutation rate spectrum becomes:

$$\mu^{(0)}_{N_1>N_2} = \mu_{N_1>N_2} \times \mu_{\mathrm{sim}}$$

where μ_{sim} corresponds to the base mutation rate per nucleotide.

Context-dependent mutation effects

CpG effect

Mutations at CpG dinucleotides (C>T and G>A) were treated as a position-specific process because of their distinct methylation-driven dynamics. This effect was empirically quantified and then modeled in the simulator using a transformed Gaussian distribution.

Empirical estimation of CpG parameters. To quantify the CpG-specific enrichment along genes, we computed position-specific mutation frequencies in a 5 kb window around each transcription start site (TSS). For each gene k and each position i, we recorded:

- the number of observed C>T and G>A SNPs, $SNP_{k,i} \in \{0, 1\}$;
- the presence of a CpG dinucleotide, $CpG_{k,i} \in \{0, 1\}$.

The total number of mutations and CpG sites at position i were:

$$SNP_i = \sum_{k=1}^{G} SNP_{k,i}, \tag{1}$$

$$CpG_i = \sum_{k=1}^{G} CpG_{k,i}. \tag{2}$$

The observed mutation frequency at position i was then:

$$[f]_i = \frac{SNP_i}{CpG_i}, \quad 0 \leq [f]_i < 1. \tag{3}$$

To correct for recurrent mutations at the same site, which lead to underestimation of the true mutation rate, we applied:

$$[f]_i^* = \frac{[f]_i}{1 - [f]_i}, \quad [f]_i^* > 0. \tag{4}$$

The resulting positional rates $[f]_i^*$ were then fitted with a Gaussian function:

$$C_{\text{CpG}}(x) = 1 + F_{\text{CpG}} \times \exp\left(-\frac{(x - L_{\text{CpG}})^2}{2\sigma_{\text{CpG}}^2}\right) + \delta_{\text{CpG}}. \tag{5}$$

Here, F_{CpG} is the amplitude, L_{CpG} the peak position relative to the TSS, σ_{CpG} the width, and δ_{CpG} a baseline offset. The fitted parameters $(L_{\text{CpG}}, F_{\text{CpG}}, \sigma_{\text{CpG}}, \delta_{\text{CpG}})$ were then used to parameterize the CpG mutation model in the simulator.

Simulation of the CpG positional effect. In the simulation, the empirically estimated double Gaussian enrichment was approximated by a transformed Gaussian-like function designed to reproduce its shape while maintaining numerical stability near the TSS. For each position x in the simulated region, the CpG mutation rate distribution is given by:

$$\mu_{\text{CpG}}(x) = (\mu_{CpG}^{(0)} - \mu_{\text{peak}}) \left[1 - \frac{\text{pdf}_{\text{trans}}\left(\frac{x}{\sigma_{\text{CpG}}},\, \epsilon,\, \delta_{\text{CpG}}\right)}{\text{pdf}_{\text{trans}}(0,\, \epsilon,\, \delta_{\text{CpG}})}\right] + \mu_{\text{peak}}, \tag{6}$$

where $\mu_{CpG}^{(0)}$ is the baseline mutation rate of $C > T$ and $G > A$ mutations, μ_{peak} the maximal mutation rate at the TSS, and σ_{CpG}, δ_{CpG} are parameters controlling the width and curvature of the curve. The function $\text{pdf}_{\text{trans}}$ applies a hyperbolic transformation to the standard normal PDF.

This transformed Gaussian formulation produces a smooth, strand-symmetric double-peak enrichment pattern around the TSS that closely matches empirical CpG mutation profiles while being computationally efficient for large-scale simulations.

Regional adaptive constraints

Regional effects represent adaptive constraints on mutation and were implemented as multiplicative scalars applied to the baseline mutation matrix. These effects reduce mutation rates in coding and untranslated regions, reflecting purifying selection on functional elements. Empirically determined constants from ENCODE [The ENCODE Project Consortium, 2007] were applied:

$$f_{\text{UTR}} = 0.7, \quad f_{\text{CDS}} = 0.2,$$

multiplicatively to sites annotated as 5'UTR or CDS, respectively.

Position-dependent modifiers. To capture finer-scale variation around splice sites and the TSS, one additional smooth position-dependent functions were fitted to the observed nucleotide frequencies, common to the 12 mutation types:

$$f_{\text{Exon}}(x) = d + a_1 e^{-\frac{(x-b_1)^2}{2c_1^2}} + a_2 e^{-\frac{(x-b_2)^2}{2c_2^2}}, \tag{7}$$

$$f_{\text{Intron}}(x) = \frac{1}{1-(ae^{-bx}+c)}. \tag{8}$$

The first function describes the TSS domain (double Gaussian), and the second describes the exon/intronic region (reciprocal function). These modifiers account for the shared positional effects acting on all mutation categories.

Residual corrections. In addition to these shared positional modifiers, we tested additional mutation-specific residual corrections. To determine whether adding such a residual correction significantly improved the model fit in these regions, we compared models with and without the correction for the different mutation types using the Akaike Information Criterion (AIC).. The log-likelihood ($\log L$) of the observed data (**O**) given the simulated results (mean $\boldsymbol{\mu}$ and variance $\boldsymbol{\sigma}^2$), assuming a Gaussian error model, was computed for each base ($i \in \{A, C, G\}$) using the following equation:

$$\log L_i = -\frac{1}{2} \sum_j \left(\frac{(O_{i,j} - \mu_{i,j})^2}{\sigma_{i,j}^2} + \ln(2\pi\sigma_{i,j}^2) \right) \tag{9}$$

where $O_{i,j}$ is the j-th observed value for base i, and $\mu_{i,j}$ and $\sigma_{i,j}^2$ are the corresponding simulated mean and variance, respectively. The sum is taken over all observation points j.g-likelihood ($\log L$) of the ob The overall log-likelihood ($\log L_{total}$) was taken as the sum of the log-likelihoods across the considered bases (e.g., $\log L_{total} = \log L_A + \log L_C + \log L_G$). The AIC was calculated as:

$$\text{AIC} = 2k - 2 \cdot \log L_{total} \tag{10}$$

where k is the number of parameters in the model. The model leading to the minimum AIC value was considered the best fit to the empirical data and we thus considered their residual functions to model the mutation rates across sites.

GC-biased gene conversion (gBGC)

GC-biased gene conversion was implemented as a fixation bias acting during recombination. The population-scaled intensity B modulates the relative fixation probabilities of weak-to-strong (WS) and strong-to-weak (SW) mutations:

$$p_{\mathrm{WS}} = \frac{1 - e^{-2b}}{1 - e^{-4N_e b}}, \qquad p_{\mathrm{SW}} = \frac{1 - e^{2b}}{1 - e^{4N_e b}}, \tag{11}$$

where $b = B/(4N_e)$ and $N_e = 10{,}000$ is the effective population size. The fixation bias relative to neutrality is expressed as

$$f_{\mathrm{WS}} = \frac{p_{\mathrm{WS}}}{p_{\mathrm{WWSS}}}, \quad f_{\mathrm{SW}} = \frac{p_{\mathrm{SW}}}{p_{\mathrm{WWSS}}}, \quad p_{\mathrm{WWSS}} = \frac{1}{2N_e}.$$

Two components of gBGC are considered:

1. a **background effect** (B_{b}) applied uniformly across the genome, and
2. an **additional effect** (B_{add}) centered around the TSS when subjected to hotspot of recombination.

The total bias at TSS is $B_{\mathrm{TSS}} = B_{\mathrm{b}} + B_{\mathrm{add}}(x)$, where the hotspot component follows a Gaussian-like positional distribution parameterized by a peak location, amplitude, and width $(L_{\mathrm{GC}}, F_{\mathrm{GC}}, \sigma_{\mathrm{GC}})$.

At each position, WS and SW substitution rates are multiplied by the corresponding factors $f_{WS}(x)$ and $f_{SW}(x)$, thus integrating gBGC into the full mutation rate matrix.

Per-position mutation rate matrix

For each sequence of length L, the simulator constructs a mutation rate matrix

$$\mathbf{M} \in \mathbb{R}^{L\times 14},$$

where each row corresponds to one genomic position and the 14 columns represent the twelve possible base substitution types (excluding identities) plus two CpG-specific transitions (C>T and G>A at CpG sites). Each entry of this matrix corresponds to a per-generation mutation probability for a given position and substitution type, such that

$$\mathbf{M}[x,\, N_1 \to N_2] = \mu_{x,\, N_1 \to N_2}.$$

The mutation probability at position x for substitution $N_1 \to N_2$ is computed as:

$$\mu_{x,\, N_1 \to N_2} = \mu^{(0)}_{N_1 \to N_2} \times f_{\mathrm{region}}(x,\, N_1 \to N_2) \times f_{\mathrm{gBGC}}(x,\, N_1 \to N_2), \tag{12}$$

where $\mu^{(0)}_{N_1 \to N_2}$ is the baseline mutation rate for this substitution type, f_{region} represents the regional profile (f_{Exon}, f_{Intron}, f_{UTR} or f_{CDS}), f_{gBGC} accounts for GC-biased gene conversion effects (f_{WS} or f_{SW} which exact value at position x depend of the background or additional

effect), the CpG-specific mutation rate is encoded because we have 14 mutation types per position.

For some mutation types and genomic contexts, an additional multiplicative correction is applied to account for residual deviations between observed and modelled substitution rates. These corrections are implemented as per-mutation and per-position scaling factors. In practice, this means that the regional term in Equation (12) is replaced by a residual-adjusted profile:

$$f_{\text{region}}(x,\, m) \longrightarrow \begin{cases} f_{\text{region}}(x,\, m) \times f_{\text{corr}}(x,\, m) & \text{if a residual correction is defined for mutation type } m, \\ f_{\text{region}}(x,\, m) & \text{otherwise,} \end{cases}$$

where $f_{\text{corr}}(x,\, m)$ is the residual correction factor. For TSS, the $G \to A$ correction function was includeed after the AIC test. For exon-intron boudaries, the $C \to A, T \to A$ and $G \to C$ were included. Those corrections were parameterized as local Gaussian or exponential deviations centered around functional landmarks (TSS or exon-intron boundary respectively), with parameters given in Supplemental Figures 1 and 6.

Simulation of exon structure and sequence motifs

In addition to the per-position mutation rate matrix, the simulator explicitly models the structural and compositional organization at the start of genes.

Exon length distribution. The length of the first exon (distance between the transcription start site, TSS, and the 5' splice site, SS) was simulated from a generalized gamma distribution fitted to human exon 1 length data ($a = 56.1, c = 0.1966, \text{loc} = 1.876, \text{scale} = 3.479 \times 10^{-7}$; [Movassat et al., 2019]). This distribution reproduces the characteristic short and right-skewed exon length profile observed in human transcripts.

Transcription start site motif. The nucleotide at the TSS was drawn from the empirical base frequencies observed around human TSSs [Frith et al., 2008], showing a strong enrichment for adenine. In practice, the base at position 0 was sampled from weighted probabilities $[A, C, G, T] = [0.474, 0.123, 0.378, 0.026]$.

Splice site consensus. The 5' splice site sequence (10-12 bp) was generated according to a probabilistic consensus model following figure 5 of [Sibley et al., 2016]. Two classes of motifs were implemented with relative frequencies proportional to their empirical occurrences (53.6% and 45.1%), each represented by a motif with nucleotides probabilities that reproduce the nucleotide preferences of these motifs.

Start codon placement. The position of the start codon (ATG) within the first exon was drawn from a generalized gamma distribution describing 5'UTR length variation ($a = 1.795, c = 1.048, \text{loc} = 1.792, \text{scale} = 136.3$; [Leppek et al., 2018]). When the sampled start codon overlapped with the splice site, a new position was resampled to maintain structural consistency.

Sequence constraints. Once these structural elements were generated, the simulator overwrote the corresponding positions in the evolving sequence to enforce the selected motifs in place: the chosen base at the TSS, the canonical splice site motif at the 5'SS, and the ATG codon at the start of the coding region. Mutation rates in these regions were subsequently modified according to their annotation (UTR, CDS, or intronic) as described above.

Forward-in-time sequence evolution

The simulator evolves sequences forward in time under these position-dependent rates. At initialization, each position is assigned a nucleotide according to the stationary distribution derived from the baseline mutation matrix ($Q\pi = 0$).

Each evolutionary round corresponds to one complete update over all L positions. During a round, every site can mutate with a probability given by its current base-specific rate, conditional on its present nucleotide. This represents one full generation cycle of potential substitution events across the sequence.

The absolute timescale of a round (T_{round}) is calibrated from the ratio of the simulated to real mutation rates:

$$T_{\text{round}} = \frac{\mu_{\text{sim}}}{\mu_{\text{real}}},$$

with $\mu_{\text{sim}} = 0.5 * 10^{-3}$ per round, $\mu_{\text{real}} = 2 \times 10^{-8}$ per generation. Thus, one simulated round corresponds to approximately $\approx 27,000$ generations.

Two successive evolutionary phases are simulated:

1. a **phase with active hotspot gBGC** ($X = 500$ rounds, $B_{\text{TSS}} = B_{\text{add}} + B_b$), followed by
2. a **phase after hotspot deactivation** ($Y = 100$ rounds, $B_{\text{TSS}} = B_b$), where only background gBGC and adaptive constraints remain.

This allows testing whether an ancestral hotspot gBGC is necessary to reproduce the observed patterns.

Model fitting and comparison

Simulated base composition profiles were compared to the empirical mutation rate and GC content profiles estimated around TSS. For each simulated parameter combination, a Relative Mean Squared Deviation (RMSD) score was computed to quantify the distance to the empirical data.

For a given base $i \in \{A, C, G\}$ and a simulation run s, the deviation ($\Delta_{i,s}$) was calculated across all observation points j as the sum of the squared relative differences between the simulated value ($\text{Sim}_{i,j,s}$) and the corresponding observed real value ($O_{i,j}$).

$$\Delta_{i,s} = \sum_j \left(\frac{\text{Sim}_{i,j,s} - O_{i,j}}{O_{i,j}} \right)^2 \tag{13}$$

The overall score for the simulation run s (Score_s) was then determined by summing the deviations across the considered bases i:

$$\text{Score}_s = \Delta_{A,s} + \Delta_{C,s} + \Delta_{G,s} \tag{14}$$

The nucleotide T was excluded since base frequencies sum to 1. The configuration with the lowest Score was taken as the overall best-fitting model, indicating the best agreement between the simulation results and the empirical data..

Implementation and reproducibility

All analyses were implemented in Python using standard scientific libraries (numpy, scipy, pandas, and matplotlib). The first script, *mutation_rate.py*, provides functions to generate per-position mutation rates, including regional effects (TSS, EIB), CpG-specific transitions, residual corrections, and gBGC factors. The second script, *sequences_generation.py*, generates nucleotide sequences at equilibrium using these mutation rates and implements in-place mutations, consensus splice sites, and sequence constraints. The third script, *simulator_gc_tss_mr_graph.py*, integrates the previous modules and produces the final per-position mutation rate matrixand run the simulations.

Table 1: Correspondence between the manuscript notation, the concepts, and the Python implementation.

Symbol / Concept	Code variable / function	Description
M	*mut_rate_matrix*	Per-position mutation rate matrix; rows = 14 substitutions (including CpG), columns = sequence positions.
$\mu^{(0)}_{N_1 \to N_2}$	*mutation_rate[idx, N[mut_name]]*	Baseline mutation rate for each substitution type and position.
f_{region} (TSS/EIB)	*TSS_mutation_rate()*, *EIB_mutation_rate()*	Regional modifiers for mutation rates along the sequence.
Residual correction	*residual_TSS()*, *residual_EIB()*	Correction applied to regional effects depending on mutation type.
gBGC background	*background_gBGC()*	Modifies mutation rates genome-wide according to background GC-biased gene conversion.
gBGC hotspot (TSS)	*hotspot_gBGC()*	Applies local gBGC scaling to TSS regions.
Equilibrium probabilities	*compute_equilibrium_probs()*	Solves per-position transition matrix to obtain equilibrium nucleotide probabilities.
Sequence generation	*newsequence_equilibrium()*	Generates a sequence according to equilibrium probabilities and applies regional, residual, CpG, and gBGC effects.
Mutation for one round of evolution	*mutate_inplace()*	Applies mutations directly to a sequence using the prepared per-position mutation probabilities.
Splice motifs	*consensus_splice_bytes()*, *conserve_inplace()*	Generate and fix consensus splice site motifs in the sequence.

The code is available in the project repository (`https://github.com/tinaqiu221/TSS_hypermutation/tree/main/Nucleotide_evolution_simulator`).

References

[Frith et al., 2008] Frith, M. C., Valen, E., Krogh, A., Hayashizaki, Y., Carninci, P., and Sandelin, A. (2008). A code for transcription initiation in mammalian genomes. *Genome Research*, 18(1):1–12.

[Leppek et al., 2018] Leppek, K., Das, R., and Barna, M. (2018). Functional 5' UTR mRNA structures in eukaryotic translation regulation and how to find them. *Nature Reviews Molecular Cell Biology*, 19(3):158–174.

[Movassat et al., 2019] Movassat, M., Forouzmand, E., Reese, F., and Hertel, K. J. (2019). Exon size and sequence conservation improves identification of splice-altering nucleotides. *RNA*, 25(12):1793–1805.

[Sibley et al., 2016] Sibley, C. R., Blazquez, L., and Ule, J. (2016). Lessons from non-canonical splicing. *Nature Reviews Genetics*, 17(7):407–421.

[The ENCODE Project Consortium, 2007] The ENCODE Project Consortium (2007). Identification and analysis of functional elements in 1% of the human genome by the ENCODE pilot project. *Nature*, 447(7146):799–816.
