# Supplementary material for: Non-adaptive evolutionary forces predominantly shape nucleotide composition near human transcription start sites
Source: PLoS Biol. 2026 Oct 21;24(10):e3004063. doi: 10.1371/journal.pbio.3004063 (PMC13712754; doi:10.1371/journal.pbio.3004063)
Supplement: Supplementary file 2 [file pbio.3004063.s010.docx]

| **Mutation dataset** | **Number of mutations** |
| --- | --- |
| gnomAD rareSNP (<1%) | 503,266,121 |
| gnomAD rareSNP (<0.1%) | 468,914,883 |
| DNM | 1,083,560 |
| Somatic mutations | 3,487,631 |

**S1 Table. Number of mutations/SNPs per dataset.**
