# Supplementary material for: Non-adaptive evolutionary forces predominantly shape nucleotide composition near human transcription start sites
Source: PLoS Biol. 2026 Oct 21;24(10):e3004063. doi: 10.1371/journal.pbio.3004063 (PMC13712754; doi:10.1371/journal.pbio.3004063)
Supplement: Supplementary file 3 [file pbio.3004063.s011.docx]

| **DNM source** | **Number of unique trios/probands (after removal of shared variants)** | **Number of mutations (after removal of shared variants)** |
| --- | --- | --- |
|  |  |  |
| An et al, 2018 | 3804 | 232327 |
| Francioli et al., 2015 | 258 | 11000 |
| Goldmann et al., 2016 | 816 | 35702 |
| Palsson et al., 2025 | 9651 | 606438 |
| Richter et al., 2020 | 763 | 54070 |
| Sasani et al., 2019 | 420 | 27634 |
| Yuen et al., 2017 | 1627 | 116389 |

**S2 Table. DNM sources, number of trios and mutations.**
