# Supplementary material for: Non-adaptive evolutionary forces predominantly shape nucleotide composition near human transcription start sites
Source: PLoS Biol. 2026 Oct 21;24(10):e3004063. doi: 10.1371/journal.pbio.3004063 (PMC13712754; doi:10.1371/journal.pbio.3004063)
Supplement: Supplementary file 4 [file pbio.3004063.s012.docx]

| **DAF bin** | **Intergenic** | **TSS -150 to +350** | **Exon (TSS 0 to +350)** | **Intron (TSS 0 to +350)** | **Recombination hotspot** |
| --- | --- | --- | --- | --- | --- |
| **0-0.001** | 0.004% | 0.008% | 0.007% | 0.007% | 0.004% |
| **0.001-0.01** | 0.47% | 0.41% | 0.34% | 0.46% | 0.48% |
| **0.01-0.1** | 3.84% | 3.41% | 3.59% | 3.04% | 3.79% |
| **0.1-0.5** | 24.44% | 23.99% | 23.22% | 23.80% | 25.17% |
| **0.5-0.9** | 66.46% | 66.32% | 66.70% | 65.30% | 67.30% |
| **0.9-1** | 98.98% | 99.10% | 99.35% | 98.73% | 98.68% |

**S3 Table. Mispolarization rate of gnomAD reference/alternate alleles across derived allele frequency bins for each analyzed genomic region.**
