# Supplementary material for: Non-adaptive evolutionary forces predominantly shape nucleotide composition near human transcription start sites
Source: PLoS Biol. 2026 Oct 21;24(10):e3004063. doi: 10.1371/journal.pbio.3004063 (PMC13712754; doi:10.1371/journal.pbio.3004063)
Supplement: Supplementary file 8 [file pbio.3004063.s017.docx]

Dear Richard Hodge,

Below is our point-by-point reply to the reviewers’ concerns. Our responses are in red.

Thank you,

Alex Palazzo

REVIEWS:

Reviewer #1: The revised manuscript has improved substantially, and my concerns have been addressed. However, I believe that the following issues still require attention.

We sincerely thank the reviewer. Their comments alerted us to a few minor problems that we have now fixed and have improved the manuscript.

1. Something appears to be incorrect in Supplemental Figure 1B. The average mutation rate predicted from the 5-mer model is of approximately the same magnitude as the rate of the most highly mutable CpG>TpG mutation class. This seems implausibly high for an average across all mutation types and suggests a possible error in the normalization by sequence-context opportunities.

The authors argue that the high predicted rate is expected because CpGs near TSSs are hypomethylated and therefore experience lower mutation rates than CpGs in the intergenic regions used to estimate the context-specific rates. Although this may contribute to the discrepancy, it does not appear sufficient to explain its magnitude. The authors should carefully verify the calculation, particularly the denominator used to convert the weighted 5-mer values into an expected per-site mutation rate, and provide a more quantitative explanation if the result is correct.

We thank the reviewers for pointing out this issue. Indeed, the magnitude of the high context predicted rate for mutations including CpGs was artificially inflated due to an incorrect odds transformation. CpG contexts are almost entirely mutated (*p* ≈ 0.85-0.95) in the intergenic calibration. We previously applied the odds transformation $p/\left( 1-p \right)$, which is appropriate for logistic-regression-style modeling but not for converting a saturating polymorphism proportion back into an underlying mutation rate. We now instead apply $-\ln\left( 1-p \right)$, a transformation that treats mutation accumulation as a Poisson process. We corrected this for all *M* calculations and regenerated all of the figures where *M* is graphed.

2. The mutational-signature analysis is considerably clearer in the revised manuscript. However, there still appears to be a problem with the normalization for trinucleotide opportunities.

The Methods state that the authors either fit the raw mutation counts directly or divide both the observed mutation spectrum and the COSMIC reference signatures by the trinucleotide composition of each genomic window. Neither procedure provides the appropriate correction for differences between the trinucleotide composition of the analyzed window and that of the whole genome.

COSMIC signatures are defined relative to the trinucleotide opportunities of the reference genome. Therefore, before applying NNLS to a mutation spectrum from a specific genomic window, the observed counts should first be divided channel-wise by the trinucleotide opportunities in that window and then multiplied channel-wise by the corresponding whole-genome trinucleotide opportunities. The resulting opportunity-adjusted spectrum can then be fitted using the unmodified COSMIC signatures. Equivalently, the COSMIC signatures could be transformed to the local opportunity composition, but only through the corresponding ratio of local to genome-wide opportunities. Dividing both the observed spectra and the COSMIC signatures by the local trinucleotide frequencies does not achieve this correction and may distort the inferred signature activities.

We thank the reviewer for pointing out this issue. We agree that our previous normalization did not correctly account for differences between the trinucleotide composition of each genomic window and that of the reference genome.

We revised the mutational-signature analysis with the proper normalization. For each genomic window, we now calculate the number of available sites for each trinucleotide directly from the reference genome sequence. We also calculate the corresponding trinucleotide opportunities across the whole reference genome. We then correct the observed mutation spectrum using the ratio of genome-wide to local trinucleotide opportunities:

**adjusted mutation count = observed mutation count × (genome-wide opportunity / local opportunity)**

The resulting opportunity-adjusted mutation spectrum is then fitted using the original, unmodified COSMIC signatures.

We have also removed the previous normalization procedure in which both the observed spectrum and the COSMIC signatures were divided by the local trinucleotide frequencies.

The new SBS signatures are present in Supplemental Figure 2. The results now look much cleaner than before and we sincerely thank the reviewer for helping us with this analysis.

3. The following sentence from the Discussion section should be corrected by replacing "low frequency" with "high-frequency":

"Indeed, selection should be primarily acting to purge deleterious mutations and thus suppressing variation in the low frequency SNP pool."

We are sorry for not being clear. We now rewrote this sentence: “If selection were significantly altering the makeup of this pool, it would mostly purge deleterious mutations and thus suppress variation.”


Reviewer #2 (Sheel Chandra, identifies herself): I thank the authors for testing the concerns raised in the review. I still have two outstanding questions/concerns.

As with the first reviewer, we sincerely thank you as your numerous comments (in this round and previous rounds) prompted us to reanalyze the data, making it cleaner, and even more compelling. In light of this, we now expressly thank you and the other reviewer in the Acknowledgements.

1) Thank you for adding Supp Fig 4A-B. However, I don't think this fully addresses my original concern. My comment raised two possible explanations for the discrepancy with Guzman et al. (2025)'s observation: (1) different DNM datasets, and (2) Guzman et al.'s use of sequence-context correction. The new analysis tests something adjacent to (1), but it looks at the Seplyarskiy et al. (2023) dataset rather than the actual Guzman et al. dataset. Since Guzman et al.'s dataset (they report 774,930 DNMs from a 10-study meta-cohort) is larger than Palsson et al.'s, this actually suggests power may not explain the discrepancy. This brings us to (2). I'd find it valuable if the authors could repeat the DNM analysis with sequence-context correction, as they did for the rare-variant analysis, and report whether the hypermutation signal persists. I think this is worth clarifying, since it bears on whether the signal reflects a germline process as the paper concludes.

We thank the reviewer for prompting us to reanalyze *de novo* mutations with the increased set and comparing it to the context. The new de novo mutation set increased to over 1.08x10^6^ SNVs. Using this new set we have now updated Figure 3C-D and Supplemental Figure 4A-B. We also updated the discussion of the discrepancy between our results and Cortés Guzmán et al.

2) I thank the authors for now polarizing mutations using the ancestral genome and for including the SFS analysis. I remain concerned, however, that the substitution rates underlying the equilibrium analysis still rely on ancestral allele polarization based on outgroup comparison. As noted, the chance that the ancestral state differs from the outgroup is higher at hypermutable sites such as TSS. Have the authors considered re-deriving these substitution rates using a probabilistic ancestral state reconstruction? Alternatively, they could use tools such as Progressive Cactus (Armstrong et al., 2020), which builds a reference-free multiple alignment based on multiple species and infers ancestral sequence at each internal node.

Again, we thank the reviewers for prompting us to reanalyze these data. We recalculated substitution rates using the inferred ancestral sequence from ENSEMBL that was derived from a probabilistic reference-free ancestral state reconstruction (Paten et al., 2008). We obtained similar findings. As this is a very important control, we now include it in Supplemental Figure 6.
