# Supplementary material for: Non-adaptive evolutionary forces predominantly shape nucleotide composition near human transcription start sites
Source: PLoS Biol. 2026 Oct 21;24(10):e3004063. doi: 10.1371/journal.pbio.3004063 (PMC13712754; doi:10.1371/journal.pbio.3004063)
Supplement: Supplementary file 11 [file pbio.3004063.s015.docx]

**Manuscript number:** RC-2025-03317

**Corresponding author(s):** Alexander Palazzo

## 1. General Statements

We are pleased to submit our revised manuscript, **Transcription-induced mutation and ancient gBGC shape the evolution of human transcriptional start sites**, that we plan to transfer to *PLOS Biology*.

This manuscript presents the first single-nucleotide-resolution map of germline mutation rate variation surrounding human transcription start sites, revealing a striking hypermutation hotspot driven by RNA Polymerase II activity in the testes. We then estimate how local recombination impacts these same regions to increase GC-content through GC-biased gene conversion (gBGC), and distinguish the contributions of ongoing background gBGC from ancestral TSS-associated episodes. Finally, we develop a forward-in-time simulation algorithm that integrates localized mutation rates with varying levels and patterns of gBGC to model the evolution of nucleotide composition around the TSS. Together, these analyses demonstrate that the nucleotide landscape surrounding human protein-coding gene starts is largely shaped by non-adaptive forces (mutation bias and gBGC) rather than natural selection alone.

In response to the reviewers' comments, we have made several substantial additions that have strengthened our study. Most notably, we have added a site frequency spectrum analysis across derived allele frequency bins, with proper ancestral allele polarization, which directly demonstrates that the elevated GC-content near the TSS is not maintained by ongoing selection or gBGC, but is instead consistent with ancestral gBGC (Figure 4G-H). This solves many issues that were present in Figure 5 and Supplemental Figure 4 from the previous manuscript. As such, we no longer include these Figures in the revised version. We also compare our M results to expected M data based on 5-mer sequence context (Figure 1C, Supplemental Figures 1B, 4D-H), confirming that the TSS hypermutation signal exceeds what is predicted by local pentanucleotide composition alone. We have excluded CpG sites from our equilibrium analyses (Figure 4A-F) to avoid confounding by methylation-dependent dinucleotide effects. We also include new analyses of lncRNA TSSs not expressed in testes (Figure 3), somatic mutations from SomaMutDB (Supplemental Figure 1E-F), and expanded SBS signature characterization across sub-regions of the TSS (Supplemental Figure 2). Gene/region and mutation counts are now reported in Figure 1 and Supplemental Table 1.

We believe this work is well suited for *PLOS Biology*, as it integrates population genomics, evolutionary modeling, and fine-scale genome annotation in a way that aligns with the journal's scope and readership. The manuscript extends findings from our previous study that went through the Review Commons process and was published (Qiu et al., *Genome Biology* 2024, 25:219).

## 2. Point-by-point description of the revisions

**Here is a point-by-point response to the two reviewers’ concerns. Our responses are in red.**

**Sincerely,**

**Alexander Palazzo**

Reviewer #1 (Evidence, reproducibility and clarity (Required)):

In this study, the authors identify an elevated human germline mutation density centered on transcription start sites (TSSs) of protein-coding genes and lncRNAs, using rare variants from a large sequencing cohort. The hotspot correlates with testis expression and is mirrored at intergenic RNA polymerase II pause sites, supporting a transcription-associated mechanism. The manuscript further investigates how gBGC interacts with these mutational processes to shape nucleotide composition around TSSs. Overall, the results are consistent with recent related work cited by the authors [1] and add an estimate of gBGC strength in the TSS vicinity. The general approach is methodologically strong, but several aspects raise concern about potential confounding by CpG context.

We thank the reviewer for their kind words. We tried our best to address all of the concerns.

Major comments

1. The mutational signatures inferred for the hotspot are interesting, but also partly unexpected. When refitting COSMIC-like signatures, did the authors renormalize their inputs to the appropriate mutational target sizes (i.e., the trinucleotide context frequencies of the genomic regions being analyzed)?

We now perform the COSMIC SBS analysis in 50bp windows throughout the entire 1kb regions of interest, both normalizing by the trinucleotide frequencies and without normalization (Supplemental Figure 2). When analyzing DNMs from parent offspring trio analysis, the SBS signatures became extremely variable and changed drastically depending on the window size chosen, likely due to the paucity of data. Since this data set produced unreliable results, we excluded it.

In light of earlier studies, SBS98 may reflect similarity to germline signature component 11 from Seplyarskiy et al. [2]; SBS23 appears comparable to SBS11 and other C>T-rich signatures reported at TSSs [1]; and SBS39 (C>G-dominated) is matching the finding in Cortes et al. and resembles germline component 8 [1,2]. An association of the SBS39 C>G mutation pattern with homologous recombination deficiency was also found in RefSig 3 of Degasperi et al. [3]. It would be good to put the signatures identified here in these already established contexts.

We are grateful to the reviewer for prompting us to examine the SBS signatures more closely. We now discuss our SBS signatures with reference to all of the above.

2. A major point concerns the magnitude of the hotspot relative to the "random intergenic" baseline in Fig. 1, which appears larger than previously reported for the subset of non-CpG mutations [1]. This is surprising for several reasons: (i) CpGs are included here, yet CpG mutation density is reduced at the TSS (Fig. 1E);

We agree that this is a major concern. We now include separate +/- CpG data for almost all regions analyzed.

(ii) the allele-frequency cutoffs used (0.1%-1%) include older variants, and the hotspot signal was shown to decay with variant age;

We see a slight decrease in mutations when *M_1%_* is compared to *M_0.1%_* (Figure 2D) and thus our estimates, if anything likely underrepresents the true mutation rate. And as you note, Cortés Guzmán et al. find that the levels go down with higher frequencies.

and (iii) background mutation rates in intergenic regions are often higher than in promoter-proximal sequence due to factors such as replication timing. Some of this discrepancy may reflect the assumption that Gx (the mean residence time in the DAF bin) is the same in intergenic and TSS-proximal regions.

We thank the reviewers for pointing this out, and we now state ”The number of these low frequency variants depends not only on the mutation rate (*µ*) but also on population genetics factors such as the effective population size (*N_e_*), the selection coefficient (*s*) and the conversion bias due to gBGC (*b*) which affect not only the probability to observe these mutations **but also their residence time in the 1% frequency bin**”. Despite these issues, when we compare our results to *de novo* mutations from parent-offspring trio-sequencing, we see that the intergenic mutation rate is slightly lower than the region around the TSS (Figure 3C), so we are confident that our metric M is not too far away from the real mutation rate value.

In addition, the intergenic baseline may not be an appropriate counterfactual because it is not matched to promoter/TSS sequence context. Mutation rates depend strongly on local context, therefore differences between TSS windows and random intergenic regions can reflect opportunity structure rather than per-opportunity mutagenesis. A more informative baseline would predict the expected TSS mutation profile by weighting intergenic context-specific rates (ideally trinucleotide, or at minimum CpG vs non-CpG) by the context opportunities in each TSS bin.

We now compare our *M* values to the expected context rate by computing the mutation rate for all 5-mers in intergenic regions and computing the expected rate for all regions examined (Figure 1C, Supplemental Figures 4B-F).

More broadly, CpG mutations present multiple challenges (recurrence, methylation dependence, dinucleotide context, multiple hits, polarization/ILS) that may influence several analyses. The authors implicitly acknowledge this in their discussion of Supplemental Fig. 7, where CpG mutations are excluded to compare M1% to M0.1%, consistent with the known impact of recurrent CpG>TpG mutations on DAF [4]. Given this sensitivity, it would strengthen the manuscript to apply the same CpG exclusion strategy used in Fig. 5 to additional figures, including Fig. 1. Repeating key analyses using DNMs (rather than rare variants) would further help isolate recurrence-driven effects.

We appreciate the reviewer’s comment and we now provide a more comprehensive analysis. As described above, we have tried our best to provide all data +/- CpGs. As for DNM, we have found that most of the trends are still present (see Figure 3G), however the paucity of data limits our ability to reproduce all of our findings with this dataset.

3. The CpG issue is particularly relevant for Fig. 4, which relies on a mononucleotide Markov model to infer equilibrium base composition. Because CpG effects are inherently dinucleotide- and methylation-dependent, a mononucleotide model may be substantially confounded unless CpGs are excluded or modeled explicitly.

We now compute this data without CpGs. Note that we also extended the area of analysis from 1kb to 5kb (Figure 4E-F) and discovered a broad shallow peak (Figure 4E) that was not visible in our prior analysis. We now discuss the limitations of excluding CpGs in this analysis and provide a rationale for our *in silico* modeling, which does take into account CpG mutations.

Relatedly, it is not fully clear from the Methods whether mutation/substitution rates were normalized by the correct number of mutational opportunities. For example, if πC is derived from terms like μT→C/(μT→C+μC→T), then estimating μX→Y by dividing counts of X→Y events only by bin length (25 bp) rather than by the number of X-sites (opportunities) can bias equilibrium estimates, particularly in CpG-island promoters where GC content is high. This could provide an alternative explanation for discrepancies between observed GC content and GC content predicted from substitution rates.

Thank you for pointing this out. Indeed, the mutation rates were normalized by the number of opportunities, and this is now explicitly stated in the methods.

4. To complement the equilibrium-based analyses of Fig. 4, it is further recommended testing for a TSS-localized GC-favoring bias directly using WS vs SW asymmetries normalized by mutational opportunities across multiple DAF bins. Specifically, by binning sites by distance to the TSS (e.g., the existing 25-bp bins) and computing, within each bin, a polymorphism-based skew comparing the frequency distributions of WS and SW variants (normalized by the number of ancestral W vs S opportunities). A TSS-localized gBGC signal acting today should appear as a peak in this skew near the TSS, which gets stronger with increasing DAF. Optionally, a parallel divergence-based analysis could quantify a directional substitution skew (WS vs SW, opportunity-normalized) on the human and chimp branches; extending this to an explicit ancestral branch would require ancestral reconstruction (e.g., via multi-outgroup inference), but could provide a cleaner timing test ("pre- vs post-split") without invoking compositional equilibrium.

We thank the reviewer for prompting us to analyze WS vs SW asymmetry opportunities across multiple DAF bins for TSS. This now appears in Figure 4G and clearly shows no recent bias for the fixation of WS over SW, ruling out either selection for GC or gBGC acting at human TSSs in recent evolutionary history.

Minor comments

1. It is unclear how the restriction to "genes expressed in testis" is applied if the gene set is already defined using testis-specific CAGE peaks.

Genes not expressed in testes were defined using annotations from Ensembl instead of CAGE-seq. We filtered by Human Protein Atlas TPM=0, which is RNA-seq based, separate from the CAGE-based TSS mapping. We now make this clear in the methods section.

2. More generally, please report the number of genes analyzed and the number of DNMs contributing to the relevant figures/estimates.

We now provide the number of genes/regions analyzed in Figure 1, and number of mutations per dataset in Supplemental Table 1.


References

[1] Cortés Guzmán, M. et al. Transcription start sites experience a high influx of heritable variants fueled by early development. Nature communications, 16(1), 10120 (2025).
[2] Seplyarskiy, V. B. et al. Population sequencing data reveal a compendium of mutational processes in the human germ line. Science 373, 1030-1035 (2021).
[3] Degasperi, A. et al. A practical framework and online tool for mutational signature analyses show intertissue variation and driver dependencies. Nature cancer, 1(2), 249-263 (2020).
[4] Lek, M. et al. Analysis of protein-coding genetic variation in 60,706 humans. Nature, 536(7616), 285-291 (2016).

Reviewer #2 (Evidence, reproducibility and clarity (Required)):

Qiu et al. investigate the forces shaping elevated GC content (GC%) at transcription start sites (TSSs) of human protein-coding genes. Using rare (<1%) and ultra-rare (0.1%) frequency SNPs to estimate localized mutation rates (M), validated with de novo mutations (DNMs), they identify hypermutation signals around TSSs. The authors also implicate RNA Polymerase II (RNAPII) pausing by demonstrating elevated mutability at lncRNA TSSs in testis and at intergenic RNAPII pause sites. Using analytical derivation and forward in time simulations, they argue that observed GC patterns around TSSs can be recapitulated by incorporating local mutation rate bias, weak ongoing GC-biased gene conversion (gBGC), and ancient hotspot-associated gBGC.

Some technical concerns limit confidence in the main findings. First, mutation rate estimates are not adjusted for local sequence context, despite the well-established context dependence of mutability. Second, the assumption that rare variants provide a good proxy for the mutation rate is questionable, as multiple results in the manuscript indicate that these variants remain subject to selection and gBGC. Finally, ancestral state inference using an outgroup raises concerns about mispolarization, particularly in regions of elevated mutation rates, which could inflate gBGC signals. Addressing these technical concerns, reconciling discrepancies with recent work, and clarifying the relative contributions of gBGC from selection are required to strengthen the claims in this study.

We are grateful for the reviewer’s comments and have addressed them to the best of our ability.

Major Comments

Mutation rates depend strongly on local sequence context, where even the same mutation type can differ in mutability between contexts. A recent, related study from Guzman et al. (2025), which is cited in the Discussion section, also reports hypermutability at TSSs and explicitly accounts for differences in mutation rate due to local sequence context based on a 5-mer sequence context model. A similar approach will be required to disentangle the effects of transcription-related processes on the local mutation rate from that of sequence context.

We thank the reviewer for prompting us to determine the expected context rate. We now compare our *M* values to the expected context rate by computing the mutation rate for all 5-mers in intergenic regions and computing the expected rate for all regions examined (Figure 1C, Supplemental Figures 4D-H).

The authors note that some of their results are consistent with what has been reported in Guzman et al. However, they do not address the differences in results in the two studies, specifically that Guzman et al. do not detect a hypermutation signal at TSSs when using DNMs from a meta-cohort of ten published studies. It is possible that this is because Guzman et al. used a sequence context-corrected model to estimate mutation rate at TSS, or that this study by Qiu et al. uses a larger DNM dataset (Palsson et al., 2025) which gives them more power to detect a hypermutation peak. In any case, it is worth performing the analysis on the larger dataset after adjusting for sequence context; if discrepancy in results persists, the authors need to explicitly discuss this difference.

We are grateful to the reviewer for prompting us to use the bigger DNM dataset, and we now do so (see Supplemental Figure 4A-B). We now discuss differences between our results and Cortés Guzmán in the first paragraph of the discussion.

The assumption underlying the use of rare SNPs is that they are minimally affected by selection and gBBC and thus can represent mutation patterns. However, several observations in the manuscript indicate that selection and gBGC are influencing relative frequencies of these low frequency SNPs: (1) In Figure 2, the authors find a dip in the density of rare variants around the coding region upstream of the exon/intron boundary and a sharper drop at the 5' splice site. They attribute both observations to selection. If rare variants in these regions are subject to selection, how can we be certain that variants in nearby regions are neutral?

According to our calculations the difference between *M_1%_* and *M_0.1%_* is at most 1% around the TSS (Figure 1D). We thus believe that our assumption is that *M* provides a reasonable proxy for the mutation rate is well justified. If anything, selection would expect to depress the number of inferred mutations around the TSS as observed by *M*, and the height of the real hypermutation peak would be even higher. As for gBGC, we now analyze WS vs SW asymmetry opportunities across multiple DAF bins for the TSS. This now appears in Figure 4G and clearly shows no recent bias for the fixation of WS over SW around the TSS, ruling out either selection for GC or gBGC acting at humans in recent evolutionary history.

(2) The authors also note that discrepancies between SW and WS-derived estimates of the strength of gBGC (B) may arise because WS mutations are driven to higher frequency by gBGC and can thus exceed the 0.1% threshold used. This implies that gBGC influences the frequency distribution of rare variants, violating the assumption that M reflects mutation rate alone.

We are sorry that we were not clear on this point. We actually state in the text that *M* is affected by selection and gBGC and that it is only a proxy for determining the mutation rate. We have revised this statement to “The number of these low frequency variants depends not only on the mutation rate (*µ*) but also on population genetics factors such as the effective population size (*N_e_*), the selection coefficient (*s*) and the conversion bias due to gBGC (*b*) which affect not only the probability to observe these mutations but also their residence time in the 1% frequency bin.” As you rightly point out, there are other influences on *M*, but as far as we can tell, these appear to be minor, and if anything suggests that our estimate of the hypermutation peak is an underestimate.

The inference of ancestral alleles using an outgroup is problematic. This method assumes parsimony and ignores the unlikely events of more than one mutation at the same site (eg, reversal mutations, recurrent mutations). This assumption is reasonable for most sites but breaks down for hypermutable sites. As a result, the mispolarization error is higher for sites or mutation types with higher mutation rate. If TSS regions are indeed mutation hotspots, then the high likelihood of mispolarization makes the gBGC signal in this region uncertain.

We now use an SFS-based framework to estimate gBGC (Figure 4G-H) and following the reviewer’s suggestion we use the ancestral genome build from Ensembl to determine the ancestral and derived states. In addition, we used the Ensembl build to quantified mispolarization rates in gnomAD variants. We find that mispolarization rates are low for SNPs frequency <1% and do not differ substantially between TSS and intergenic regions across allele frequency bins (Supplementary Table 2). These results suggest that differences in mispolarization are unlikely to explain the observed gBGC signal in TSS regions.

If the discrepancy in genomic equilibrium detected immediately downstream of the TSS is not a technical artifact, which lines of evidence suggest that it is not due to selection instead of ancient gBGC?

We agree with the reviewer that this was unclear, we now included a new SFS analysis (Figure 4G) which directly addresses this issue. It shows no deviation in WS/SW ratios between exonic and intronic sites around the TSS, and a similar rate in intergenic regions, arguing against ongoing selection for GC. Indeed, we now discuss this in the text: "it is very unlikely that the increase in GC at the start of genes is due to selection, either in exons or at promoters, as we would expect this type of evolutionary force to be ongoing."

Minor Comments
The authors report that the CpG mutation rate around TSS is suppressed, likely because of TSS-associated hypomethylation. However, there is a small relative excess of CpG>TpG mutations just downstream of the TSS, which is notably also reported in Guzman et al. Can the authors speculate on why this is observed? It will also be helpful to examine Fig. 1E at the same resolution as other mutation types: does the CpG>TpG peak occur at the same 25bp downstream location as other mutation types?

We appreciate this suggestion and we now explicitly examine this in Supplemental Figure 1C-D.

The authors use genes not expressed in testes to show that a lack of hypermutation signal is because transcription-related mutagenesis will not impact soma-specific genes. Are there genes that are not expressed in testis and are under constraint, and thus will be expected to have lower M?

We have no reason to think that the subset of genes not expressed in testes are under more constraint than other genes. In fact, it has been documented that constraint on protein coding genes correlates with expression levels (for example, see PMID: 22416125), and is highest for housekeeping genes. If anything, genes not expressed in the testes tend to have low overall expression levels and are expected to evolve more rapidly (i.e. are under less constraint).

To further support their hypothesis of RNAPII-associated hypermutation, the authors should also examine TSSs of lncRNAs not expressed in the germline.

We thank the reviewer for this suggestion, we now include lncRNAs not expressed in testes and see that like protein-coding genes, these lack a substantial hypermutation peak based on *M* and on DNMs (Figure 3, Supplemental Figures 4G, 5).

It is unclear whether in figure 3, "all protein coding genes" are still referring to TSSs as inferred in testis as in the previous analyses or truly all protein coding genes. If it's the latter, then that needs to be mentioned in Methods.

This is indeed all protein-coding genes. This is now explicitly stated in the text.

Supplemental figure 2: The figure caption incorrectly references Figure 2E (should be 3E). Including significance will be helpful in determining the difference in M between indicated regions. To make it further comparable with Figure 3, it will be helpful to see whether there are differences in M between regions for RNAPII pause sites across mutation types.

We thank the reviewer for pointing this out and we now examine RNA polymerase II pause sites in Supplemental Figure 5.

On page 11: "lncRNA TSSs have mutational spectra that resemble what we previously documented at TSSs of protein-coding genes" I'm not sure I agree, since SBS signatures are not similar, as shown in Supplemental Table 1.

This is an important point and we now perform further analyses on the SBS signatures. We expanded the SBS analysis, looking at 50bp windows throughout the entire 1kb regions of interest, with and without trinucleotide frequency normalization (Supplemental Figure 2). We observe similar trends in SBS signatures between the TSSs of protein coding genes and lncRNAs. We now discuss this in the text.

On page 11: need citations after paragraph describing RNAPII mutagenicity, "One of the main reasons... can elevate double-stranded breaks"

Many of these references were in the Background section. We now rewrote this later paragraph to avoid repeating ourselves. We also updated our refences in the Background section where we discuss how RNA polymerase II pausing promotes DNA damage.
