# Supplementary material for: Non-adaptive evolutionary forces predominantly shape nucleotide composition near human transcription start sites
Source: PLoS Biol. 2026 Oct 21;24(10):e3004063. doi: 10.1371/journal.pbio.3004063 (PMC13712754; doi:10.1371/journal.pbio.3004063)
Supplement: Peer review history

# Author comment: Author response to Decision Letter 0

**DOI:** 10.1371/journal.pbio.3004063.r001

20 Jun 2026

Attachment Submitted filename: 163494_1_rebuttal_3766815_tfm43v.docx

# Editor report: Decision Letter 0

**DOI:** 10.1371/journal.pbio.3004063.r002

**Authors:** Richard Hodge

6 Jul 2026

Dear Dr Palazzo,

Thank you for submitting your revised manuscript from Review Commons entitled "Transcription-induced mutation and ancient gBGC shape the evolution of human transcriptional start sites" for consideration as a Research Article by PLOS Biology. Please accept my sincere apologies for the delay in getting back to you as we consulted with an academic editor about your submission.

Your manuscript has now been evaluated by the PLOS Biology editorial staff, as well as by an academic editor with relevant expertise *EDIT AS APPLICABLE*, and I am writing to let you know that we would like to send your submission back out to the previous reviewers at Review Commons.

However, before we can send your manuscript to the reviewers, we need you to complete your submission by providing the metadata that is required for full assessment. To this end, please login to Editorial Manager where you will find the paper in the 'Submissions Needing Revisions' folder on your homepage. Please click 'Revise Submission' from the Action Links and complete all additional questions in the submission questionnaire.

Once your full submission is complete, your paper will undergo a series of checks in preparation for peer review. After your manuscript has passed the checks it will be sent out for review. To provide the metadata for your submission, please Login to Editorial Manager (https://www.editorialmanager.com/pbiology) within two working days, i.e. by Jul 08 2026 11:59PM.

During the process of completing your manuscript submission, you will be invited to opt-in to posting your pre-review manuscript as a bioRxiv preprint. Visit http://journals.plos.org/plosbiology/s/preprints for full details. If you consent to posting your current manuscript as a preprint, please upload a single Preprint PDF.

Feel free to email us at plosbiology@plos.org if you have any queries relating to your submission.

Kind regards,

Richard

Richard Hodge, PhD

Senior Editor, PLOS Biology

rhodge@plos.org

PLOS

Empowering researchers to transform science

Carlyle House, Carlyle Road, Cambridge, CB4 3DN, United Kingdom

California (U.S.) corporation #C2354500, based in San Francisco

# Author comment: Author response to Decision Letter 1

**DOI:** 10.1371/journal.pbio.3004063.r003

7 Jul 2026

Attachment Submitted filename: full-revision v6.docx

# Peer review: Decision Letter 1

**DOI:** 10.1371/journal.pbio.3004063.r004

**Authors:** Richard Hodge

3 Aug 2026

Dear Dr Palazzo,

Thank you for your patience while we considered your revised manuscript from Review Commons entitled "Transcription-induced mutation and ancient gBGC shape the evolution of human transcriptional start sites" for consideration as a Research Article at PLOS Biology. Your revised study has now been evaluated by the PLOS Biology editors, the Academic Editor and the previous reviewers.

As you will see below, the reviewers agree that the revised manuscript has been strengthened and mostly addresses the previous comments. However, they both have some remaining concerns that should be addressed in a revised version. Reviewer #1 raises concerns with the normalization procedures for several pieces of data, while Reviewer #2 notes that their previous concerns related to discrepancies with Ref 33 have not been fully addressed. He/She suggests that the DNM analysis is repeated with sequence-context correction to see whether the hypermutation signal persists to further clarify.

In light of the reviews, we are pleased to offer you the opportunity to address the remaining comments in a revision that we anticipate should not take you very long. We will then assess your revised manuscript and your response to the reviewers' comments with our Academic Editor aiming to avoid further rounds of peer-review, although we might need to consult with the reviewers, depending on the nature of the revisions.

In addition to these revisions, you may need to complete some formatting changes, which you will receive in a follow up email. A member of our team will be in touch with a set of requests shortly. If you do not receive a separate email within a few days, please assume that checks have been completed, and no additional changes are required.

We expect to receive your revised manuscript within 2 months. Please email us (plosbiology@plos.org) if you have any questions or concerns, or would like to request an extension.

At this stage, your manuscript remains formally under active consideration at our journal; please notify us by email if you do not intend to submit a revision so that we withdraw the manuscript.

**IMPORTANT - SUBMITTING YOUR REVISION**

Your revisions should address the specific points made by each reviewer. Please submit the following files along with your revised manuscript:

1. A 'Response to Reviewers' file - this should detail your responses to the editorial requests, present a point-by-point response to all of the reviewers' comments, and indicate the changes made to the manuscript.

*NOTE: In your point-by-point response to the reviewers, please provide the full context of each review. Do not selectively quote paragraphs or sentences to reply to. The entire set of reviewer comments should be present in full and each specific point should be responded to individually.

You should also cite any additional relevant literature that has been published since the original submission and mention any additional citations in your response.

2. In addition to a clean copy of the manuscript, please also upload a 'track-changes' version of your manuscript that specifies the edits made. This should be uploaded as a "Revised Article with Changes Highlighted " file type.

*Resubmission Checklist*

When you are ready to resubmit your revised manuscript, please refer to this resubmission checklist: https://plos.io/Biology_Checklist

To submit a revised version of your manuscript, please go to https://www.editorialmanager.com/pbiology/ and log in as an Author. Click the link labelled 'Submissions Needing Revision' where you will find your submission record.

Please make sure to read the following important policies and guidelines while preparing your revision:

*Published Peer Review*

Please note while forming your response, if your article is accepted, you may have the opportunity to make the peer review history publicly available. The record will include editor decision letters (with reviews) and your responses to reviewer comments. If eligible, we will contact you to opt in or out. Please see here for more details:

https://blogs.plos.org/plos/2019/05/plos-journals-now-open-for-published-peer-review/

*PLOS Data Policy*

Please note that as a condition of publication PLOS' data policy (http://journals.plos.org/plosbiology/s/data-availability) requires that you make available all data used to draw the conclusions arrived at in your manuscript. If you have not already done so, you must include any data used in your manuscript either in appropriate repositories, within the body of the manuscript, or as supporting information (N.B. this includes any numerical values that were used to generate graphs, histograms etc.). For an example see here: http://www.plosbiology.org/article/info%3Adoi%2F10.1371%2Fjournal.pbio.1001908#s5

*Blot and Gel Data Policy*

We require the original, uncropped and minimally adjusted images supporting all blot and gel results reported in an article's figures or Supporting Information files. We will require these files before a manuscript can be accepted so please prepare them now, if you have not already uploaded them. Please carefully read our guidelines for how to prepare and upload this data: https://journals.plos.org/plosbiology/s/figures#loc-blot-and-gel-reporting-requirements

*Protocols deposition*

To enhance the reproducibility of your results, we recommend that if applicable you deposit your laboratory protocols in protocols.io, where a protocol can be assigned its own identifier (DOI) such that it can be cited independently in the future. Additionally, PLOS ONE offers an option for publishing peer-reviewed Lab Protocol articles, which describe protocols hosted on protocols.io. Read more information on sharing protocols at https://plos.org/protocols?utm_medium=editorial-email&utm_source=authorletters&utm_campaign=protocols

Thank you again for your submission to our journal. We hope that our editorial process has been constructive thus far, and we welcome your feedback at any time. Please don't hesitate to contact us if you have any questions or comments.

Best regards,

Richard

Richard Hodge, PhD

Senior Editor, PLOS Biology

rhodge@plos.org

----------------------------------------------------------------

REVIEWS:

Reviewer #1: The revised manuscript has improved substantially, and my concerns have been addressed. However, I believe that the following issues still require attention.

1. Something appears to be incorrect in Supplemental Figure 1B. The average mutation rate predicted from the 5-mer model is of approximately the same magnitude as the rate of the most highly mutable CpG>TpG mutation class. This seems implausibly high for an average across all mutation types and suggests a possible error in the normalization by sequence-context opportunities.

The authors argue that the high predicted rate is expected because CpGs near TSSs are hypomethylated and therefore experience lower mutation rates than CpGs in the intergenic regions used to estimate the context-specific rates. Although this may contribute to the discrepancy, it does not appear sufficient to explain its magnitude. The authors should carefully verify the calculation, particularly the denominator used to convert the weighted 5-mer values into an expected per-site mutation rate, and provide a more quantitative explanation if the result is correct.

2. The mutational-signature analysis is considerably clearer in the revised manuscript. However, there still appears to be a problem with the normalization for trinucleotide opportunities.

The Methods state that the authors either fit the raw mutation counts directly or divide both the observed mutation spectrum and the COSMIC reference signatures by the trinucleotide composition of each genomic window. Neither procedure provides the appropriate correction for differences between the trinucleotide composition of the analyzed window and that of the whole genome.

COSMIC signatures are defined relative to the trinucleotide opportunities of the reference genome. Therefore, before applying NNLS to a mutation spectrum from a specific genomic window, the observed counts should first be divided channel-wise by the trinucleotide opportunities in that window and then multiplied channel-wise by the corresponding whole-genome trinucleotide opportunities. The resulting opportunity-adjusted spectrum can then be fitted using the unmodified COSMIC signatures. Equivalently, the COSMIC signatures could be transformed to the local opportunity composition, but only through the corresponding ratio of local to genome-wide opportunities. Dividing both the observed spectra and the COSMIC signatures by the local trinucleotide frequencies does not achieve this correction and may distort the inferred signature activities.

The authors should therefore repeat the opportunity-normalized analysis using the appropriate transformation.

3. The following sentence from the Discussion section should be corrected by replacing "low frequency" with "high-frequency":

"Indeed, selection should be primarily acting to purge deleterious mutations and thus suppressing variation in the low frequency SNP pool."

Reviewer #2 (Sheel Chandra, identifies herself): I thank the authors for testing the concerns raised in the review. I still have two outstanding questions/concerns.

1) Thank you for adding Supp Fig 4A-B. However, I don't think this fully addresses my original concern. My comment raised two possible explanations for the discrepancy with Guzman et al. (2025)'s observation: (1) different DNM datasets, and (2) Guzman et al.'s use of sequence-context correction. The new analysis tests something adjacent to (1), but it looks at the Seplyarskiy et al. (2023) dataset rather than the actual Guzman et al. dataset. Since Guzman et al.'s dataset (they report 774,930 DNMs from a 10-study meta-cohort) is larger than Palsson et al.'s, this actually suggests power may not explain the discrepancy. This brings us to (2). I'd find it valuable if the authors could repeat the DNM analysis with sequence-context correction, as they did for the rare-variant analysis, and report whether the hypermutation signal persists. I think this is worth clarifying, since it bears on whether the signal reflects a germline process as the paper concludes.

2) I thank the authors for now polarizing mutations using the ancestral genome and for including the SFS analysis. I remain concerned, however, that the substitution rates underlying the equilibrium analysis still rely on ancestral allele polarization based on outgroup comparison. As noted, the chance that the ancestral state differs from the outgroup is higher at hypermutable sites such as TSS. Have the authors considered re-deriving these substitution rates using a probabilistic ancestral state reconstruction? Alternatively, they could use tools such as Progressive Cactus (Armstrong et al., 2020), which builds a reference-free multiple alignment based on multiple species and infers ancestral sequence at each internal node.

# Author comment: Author response to Decision Letter 2

**DOI:** 10.1371/journal.pbio.3004063.r005

31 Aug 2026

Attachment Submitted filename: Response_to_reviewers_3.docx

# Editor report: Decision Letter 2

**DOI:** 10.1371/journal.pbio.3004063.r006

**Authors:** Richard Hodge

9 Sep 2026

Dear Dr Palazzo,

Thank you for your patience while we considered your revised manuscript "Transcription-induced mutation and ancient gBGC shape the evolution of human transcriptional start sites" for publication as a Research Article at PLOS Biology. This revised version of your manuscript has been evaluated by the PLOS Biology editors and the Academic Editor.

Based on our Academic Editor's assessment of your revision, I am pleased to say that we are likely to accept this manuscript for publication, provided you satisfactorily address the following data and other policy-related requests that I have provided below (A-F):

(A) We routinely suggest changes to titles to ensure maximum accessibility for a broad, non-specialist readership. In this case, we would like to suggest a minor edit to your current title, as well as a new one, to ensure the title is as accessible to possible. Depending on the title you prefer, please ensure you change both the manuscript file and the online submission system, as they need to match for final acceptance:

“Transcription-associated hypermutation and ancient GC-biased gene conversion shape the evolution of human transcriptional start sites”

OR

“Non-adaptive evolutionary forces predominantly shape nucleotide composition near human transcription start sites”

(B) You may be aware of the PLOS Data Policy, which requires that all data be made available without restriction: http://journals.plos.org/plosbiology/s/data-availability. For more information, please also see this editorial: http://dx.doi.org/10.1371/journal.pbio.1001797

Note that we do not require all raw data. Rather, we ask that all individual quantitative observations that underlie the data summarized in the figures and results of your paper be made available in one of the following forms:

-Supplementary files (e.g., excel). Please ensure that all data files are uploaded as 'Supporting Information' and are invariably referred to (in the manuscript, figure legends, and the Description field when uploading your files) using the following format verbatim: S1 Data, S2 Data, etc. Multiple panels of a single or even several figures can be included as multiple sheets in one excel file that is saved using exactly the following convention: S1_Data.xlsx (using an underscore).

-Deposition in a publicly available repository. Please also provide the accession code or a reviewer link so that we may view your data before publication.

Regardless of the method selected, please ensure that you provide the individual numerical values that underlie the summary data displayed in the following figure panels as they are essential for readers to assess your analysis and to reproduce it:

Figure 1A-R, 2A-F, 3A-D, 3F-G, 4A-H, 5A-N, S1A-F, S2A-B, S3A-L, S4A-H, S5A-L, S6A-J, S7A-L, S8A-L

NOTE: the numerical data provided should include all replicates AND the way in which the plotted mean and errors were derived (it should not present only the mean/average values).

(C) Please also ensure that each of the relevant figure legends in your manuscript include information on *WHERE THE UNDERLYING DATA CAN BE FOUND*, and ensure your supplemental data file/s has a legend.

(D) Please note that we cannot accept sole deposition of code in GitHub, as this could be changed after publication. However, you can archive this version of your publicly available GitHub code to Zenodo. Once you do this, it will generate a DOI number, which you will need to provide in the Data Accessibility Statement (you are welcome to also provide the GitHub access information). See the process for doing this here: https://docs.github.com/en/repositories/archiving-a-github-repository/referencing-and-citing-content

(E) Please ensure that your Data Statement in the submission system accurately describes where your data can be found and is in final format, as it will be published as written there.

(F) Please ensure that you are using best practice for statistical reporting and data presentation. These are our guidelines https://journals.plos.org/plosbiology/s/best-practices-in-research-reporting#loc-statistical-reporting and a useful resource on data presentation https://journals.plos.org/plosbiology/article?id=10.1371/journal.pbio.1002128

- If you are reporting experiments where n ≤ 5, please plot each individual data point.

------------------------------------------------------------------------

As you address these items, please take this last chance to review your reference list to ensure that it is complete and correct. If you have cited papers that have been retracted, please include the rationale for doing so in the manuscript text, or remove these references and replace them with relevant current references. Any changes to the reference list should be mentioned in the cover letter that accompanies your revised manuscript.

In addition to these revisions, you may need to complete some formatting changes, which you will receive in a follow up email. A member of our team will be in touch with a set of requests shortly. If you do not receive a separate email within a few days, please assume that checks have been completed, and no additional changes are required.

We expect to receive your revised manuscript within two weeks.

To submit your revision, please go to https://www.editorialmanager.com/pbiology/ and log in as an Author. Click the link labelled 'Submissions Needing Revision' to find your submission record. Your revised submission must include the following:

- a cover letter that should detail your responses to any editorial requests, if applicable, and whether changes have been made to the reference list

- a Response to Reviewers file that provides a detailed response to the reviewers' comments (if applicable, if not applicable please do not delete your existing 'Response to Reviewers' file.)

- a track-changes file indicating any changes that you have made to the manuscript.

NOTE: If Supporting Information files are included with your article, note that these are not copyedited and will be published as they are submitted. Please ensure that these files are legible and of high quality (at least 300 dpi) in an easily accessible file format. For this reason, please be aware that any references listed in an SI file will not be indexed. For more information, see our Supporting Information guidelines:

https://journals.plos.org/plosbiology/s/supporting-information

*Published Peer Review History*

Please note that you may have the opportunity to make the peer review history publicly available. The record will include editor decision letters (with reviews) and your responses to reviewer comments. If eligible, we will contact you to opt in or out. Please see here for more details:

https://plos.org/published-peer-review-history/

*Press*

Should you, your institution's press office or the journal office choose to press release your paper, please ensure you have opted out of Early Article Posting on the submission form. We ask that you notify us as soon as possible if you or your institution is planning to press release the article.

*Protocols deposition*

To enhance the reproducibility of your results, we recommend that if applicable you deposit your laboratory protocols in protocols.io, where a protocol can be assigned its own identifier (DOI) such that it can be cited independently in the future. Additionally, PLOS ONE offers an option for publishing peer-reviewed Lab Protocol articles, which describe protocols hosted on protocols.io. Read more information on sharing protocols at https://plos.org/protocols?utm_medium=editorial-email&utm_source=authorletters&utm_campaign=protocols

Please do not hesitate to contact me should you have any questions.

Best wishes,

Richard

Richard Hodge, PhD

Senior Editor, PLOS Biology

rhodge@plos.org

PLOS

Empowering researchers to transform science

Carlyle House, Carlyle Road, Cambridge, CB4 3DN, United Kingdom

California (U.S.) corporation #C2354500, based in San Francisco

# Author comment: Author response to Decision Letter 3

**DOI:** 10.1371/journal.pbio.3004063.r007

6 Oct 2026

Attachment Submitted filename: Response_to_reviewers_3_auresp_3.docx

# Editor report: Decision Letter 3

**DOI:** 10.1371/journal.pbio.3004063.r008

**Authors:** Richard Hodge

6 Oct 2026

Dear Alex,

On behalf of my colleagues and the Academic Editor, Deepa Agashe, I am pleased to say that we can accept your manuscript for publication, provided you address any remaining formatting and reporting issues. These will be detailed in an email you should receive within 2-3 business days from our colleagues in the journal operations team; no action is required from you until then. Please note that we will not be able to formally accept your manuscript and schedule it for publication until you have completed any requested changes.

Please take a minute to log into Editorial Manager at http://www.editorialmanager.com/pbiology/, click the "Update My Information" link at the top of the page, and update your user information to ensure an efficient production process.

PRESS

We frequently collaborate with press offices. If your institution or institutions have a press office, please notify them about your upcoming paper at this point, to enable them to help maximise its impact. If the press office is planning to promote your findings, we would be grateful if they could coordinate with biologypress@plos.org. If you have previously opted in to the early version process, we ask that you notify us immediately of any press plans so that we may opt out on your behalf.

We also ask that you take this opportunity to read our Embargo Policy regarding the discussion, promotion and media coverage of work that is yet to be published by PLOS. As your manuscript is not yet published, it is bound by the conditions of our Embargo Policy. Please be aware that this policy is in place both to ensure that any press coverage of your article is fully substantiated and to provide a direct link between such coverage and the published work. For full details of our Embargo Policy, please visit http://www.plos.org/about/media-inquiries/embargo-policy/.

Thank you again for choosing PLOS Biology for publication and supporting Open Access publishing. We look forward to publishing your study.

Best wishes,

Richard

Richard Hodge, PhD

Senior Editor, PLOS Biology

rhodge@plos.org

PLOS

Empowering researchers to transform science

Carlyle House, Carlyle Road, Cambridge, CB4 3DN, United Kingdom

California (U.S.) corporation #C2354500, based in San Francisco
